# Genome-wide, evolutionary, and functional analyses of ascorbate peroxidase (*APX*) family in Poaceae species

**DOI:** 10.1590/1678-4685-GMB-2022-0153

**Published:** 2022-12-09

**Authors:** Douglas Jardim-Messeder, Andreia Caverzan, Gabriel Afonso Bastos, Vanessa Galhego, Ygor de Souza-Vieira, Fernanda Lazzarotto, Esther Felix-Mendes, Lucas Lavaquial, José Nicomedes, Márcia Margis-Pinheiro, Gilberto Sachetto-Martins

**Affiliations:** 1 Universidade Federal do Rio de Janeiro, Departamento de Genética, Rio de Janeiro, RJ, Brazil.; 2 Universidade Federal do Rio de Janeiro, Instituto de Bioquímica Médica, Rio de Janeiro, RJ, Brazil.; 3 Universidade Federal do Rio Grande do Sul, Departamento de Genética, Porto Alegre, RS, Brazil.; 4 Universidade Federal do Rio Grande do Sul, Centro de Biotecnologia, Porto Alegre, RS, Brazil

**Keywords:** Ascorbate peroxidase, Poaceae, reactive oxygen species, Ascorbate peroxidase-related, Ascorbate peroxidase-like

## Abstract

Ascorbate peroxidases (APXs) are heme peroxidases involved in the control of hydrogen peroxide levels and signal transduction pathways related to development and stress responses. Here, a total of 238 *APX*, 30 APX-related (*APX-R*), and 34 APX-like (*APX-L*) genes were identified from 24 species from the Poaceae family. Phylogenetic analysis of APX indicated five distinct clades, equivalent to cytosolic (*cAPX*), peroxisomal (*pAPX*), mitochondrial (*mitAPX*), stromal (*sAPX*), and thylakoidal (*tAPX*) isoforms. Duplication events contributed to the expansion of this family and the divergence times. Different from other APX isoforms, the emergence of Poaceae mitAPXs occurred independently after eudicot and monocot divergence. Our results showed that the constitutive silencing of mitAPX genes is not viable in rice plants, suggesting that these isoforms are essential for rice regeneration or development. We also obtained rice plants silenced individually to sAPX isoforms, demonstrating that, different to plants double silenced to both sAPX and tAPX or single silenced to tAPX previously obtained, these plants do not show changes in the total APX activity and hydrogen peroxide content in the shoot. Among rice plants silenced to different isoforms, plants silenced to cAPX showed a higher decrease in total APX activity and an increase in hydrogen peroxide levels. These results suggest that the cAPXs are the main isoforms responsible for regulating hydrogen peroxide levels in the cell, whereas in the chloroplast, this role is provided mainly by the tAPX isoform. In addition to broadening our understanding of the core components of the antioxidant defense in Poaceae species, the present study also provides a platform for their functional characterization.

## Introduction

Hydrogen peroxidase and other reactive oxygen species (ROS) are recognized as signaling molecules or secondary messengers participating in multiple signal transduction pathways, including environmental stimuli perception. In recent decades, many studies have supported the role of ROS in plant stress response pathways. The types of ROS and production sites allow flexibility and efficiency in many events related to the plant stress response, as well as in developmental processes, such as growth, cell cycle, programmed cell death, and hormone signaling (Mittler *et al.*, 2004; Foyer and Noctor, 2005; Gadjev et al., 2006; Shigeoka and Maruta, 2014; Vaahtera et al., 2014; Mittler, 2017). Due to their high reactivity, ROS also act as cytotoxic molecules, able to oxidize different biomolecules, such as proteins, lipids, and nucleic acids. Thus, during evolution, the development of efficient antioxidant systems has been essential for survival under changes in environmental conditions, particularly for plants, which due to their sessile lifestyle, are susceptible to a significant variety of biotic and abiotic stresses.

In addition to preventing the oxidative stress induced by ROS accumulation, the different antioxidant systems maintain the cellular concentration of ROS to a physiologic level necessary for events related to normal plant growth and development (Asada, 1999; Mittler, 2002; Mittler *et al.*, 2004). In photosynthetic organisms, the ascorbate peroxidase (APX) family (APX; EC 1.11.1.11) is the major component of the enzymatic antioxidant system. In plant cells, APX occurs in different subcellular compartments, such as peroxisomes, chloroplasts, mitochondria, and the cytosol, efficiently eliminating even very low levels of hydrogen peroxide using ascorbate as an electron donor. The mechanism of catalysis by ascorbate peroxidase is achieved using an oxidized Compound I intermediate, which is a transient species and contains a high-valent iron species (known as ferryl heme, Fe IV) and a porphyrin pi-cation radical. The compound I is subsequently reduced by reduced ascorbate in two sequential single electron transfer steps (Patterson et al., 1995; Jones et al., 1998). The APX activity is accomplished via the ascorbate-glutathione cycle, which uses the reduction potential of reduced glutathione to restore the oxidized ascorbate (Noctor and Foyer, 1998). 

Over the years, many studies have provided important insights into the relevance of different APX enzymes in the control of hydrogen peroxide levels and signal transduction pathways related to developmental stages and stress responses (Zhang et al., 1997; Yoshimura et al., 2000; Sato et al., 2001; Agrawal et al., 2003; Fryer et al., 2003; Menezes-Benavente *et al.*, 2004; Teixeira et al., 2006; Rosa et al., 2010; Gill and Tuteja 2010; Bonifacio et al., 2011, 2016; Caverzan et al., 2012, 2014, 2019; Wang et al., 2015, Jardim-Messeder et al., 2018). Besides its central role in the antioxidant metabolism in photosynthetic organisms, an additional function of APX has emerged. Using a combination of biochemical and genetic approaches, it was demonstrated that cytosolic APX from Arabidopsis and *Brachypodium distachyon* display coumarate 3-hydroxylase activity, participating in lignin biosynthesis (Barros et al., 2019).

Evolutionary studies have demonstrated that APX and cytochrome c peroxidase (CCP, EC 1.11.1.5) emerged from ancient bacterial catalase-peroxidases (KatGs, EC 1.11.1.21) (Zámocký et al., 2015), which exhibit both catalase and peroxidase activities. In eukaryotic organisms, APX and CCP families were independently acquired through endosymbiosis events that originated the chloroplast and mitochondria organelles (Lazarotto *et al.*, 2015). 

Ancient APX emerged in chlorophytes as a soluble enzyme target to chloroplast stroma. During land life adaptation, cytosolic and peroxisomal isoforms originated from duplication events. Additionally, the chloroplastic APX acquired an alternative splicing mechanism that originates both a soluble enzyme dual targeted to chloroplast and mitochondrion, as well as a thylakoid membrane-bound enzyme. Later, in some angiosperm groups such as Poales, Brassicales and Salicaceae, independent duplication and neofunctionalization events resulted in individual genes encoding soluble and membrane-bound isoforms (Qiu et al., 2020; Jardim-Messeder et al., 2022).

Considering the important role of APX in the control of hydrogen peroxide steady-state levels, the identification and characterization of APX-encoding genes are essential for understanding the different signal transduction pathways related to plant development and stress response. APX family members have been characterized in different species. In rice (*Oryza sativa*), there are eight members of the APX family encoding two cytosolic (cAPX), two peroxisomal (pAPX), two mitochondrial (mitAPX), and two chloroplastic (chlAPX) isoforms. In the chloroplast, there is one APX located in the stromal (sAPX) and another in the thylakoid (tAPX) (Teixeira et al., 2004, 2006; Xu et al., 2013; Wu et al., 2016). 

Rice is a monocot species, a member of the Poaceae family. The Poaceae, commonly referred to as grasses, is recognized as the most economically important plant family, providing more than 50% of all human dietary energy from cereal consumption (Shavanov, 2021). In addition, some Poaceae species have important roles as biofuel or building material sources. The cereal cultures, similar to the other crops, are highly threatened by environmental stresses, which are becoming increasingly frequent due to climate change events. Despite the economic and social importance of the Poaceae family, APX genes have only been identified and annotated in rice (Teixeira et al., 2004), maize (*Zea mays*) (Liu et al., 2012), sorghum (*Sorghum bicolor*) (Akbudak et al., 2018), and wheat (*Triticum aestivum*) (Tyagi et al., 2020). 

Previous work has demonstrated that the manipulation of APX gene expression alters plant development and stress response pathways. Among the Poaceae species, the APX family has been manipulated mainly in rice. Knocking out *OsAPX1* in rice results in normal development but increased seed abortion (Kim et al., 2015). *OsAPX2* knockout leads to a semidwarf phenotype and increased sensitivity to drought, salt, and cold stresses (Zhang et al., 2013). Similarly, the individual silencing of *OsAPX1* or *OsAPX2* genes impairs plant development. On the other hand, a normal phenotype and enhanced tolerance to toxic aluminum concentration have been verified in double-silenced plants to *OsAPX1* and *OsAPX2* (Rosa et al., 2010). In these plants, the altered expression of several genes associated with the photosynthetic process and antioxidant defense (Ribeiro *et al.*, 2012) and altered antioxidant response under salinity and osmotic stresses have also been verified (Cunha et al., 2016). In maize, the overexpression of cAPX (*ZmAPX1*) confers resistance to southern corn leaf blight in a jasmonic acid-mediated defense signaling pathway (Zhang *et al.*, 2022).

The double silencing of rice pAPX isoforms (*OsAPX3* and *OsAPX4*) led to early senescence (Souza *et al.*, 2015; Ribeiro et al., 2017), whereas the double silencing of sAPX and tAPX genes (*OsAPX7* and OsAPX8, respectively) impairs the protection of photosystem II (PSII) under MV-induced oxidative stress (Caverzan et al., 2014). The individual silencing of rice tAPX increased hydrogen peroxide led to closer stomata, and delayed germination in plants silenced to *OsAPX8* (Jardim-Messeder et al., 2018; Cunha et al., 2019). On the other hand, overexpression lines exhibited increased tolerance to bacterial pathogens (Jiang et al., 2016). In wheat, the silencing of the tAPX lowered photosynthetic carbon assimilation and reduced growth rate and seed production (Danna et al., 2003).

Despite different APX isoforms having been functionally characterized, the physiological role of mitAPX isoforms remains understudied. Previous works demonstrated that in rice plants, salt exposure increases the expression of mitAPX isoforms (*OsAPX5* and *OsAPX6*) (Lázaro et al., 2013), and similarly, a salt-tolerant wheat cultivar showed increased mitochondrial APX activity (Sairam and Srivastava 2002). Until the last decade, the contribution of mitochondrial respiration as a source of ROS production in plant cells was largely unexplored. In mitochondria, the respiratory complexes I, II, and III are regarded as the main sites of superoxide anion production (Møller, 2001; Jardim-Messeder et al., 2015), which is rapidly dismuted to hydrogen peroxide by mitochondrial superoxide dismutase. Due to the absence of catalase in plant mitochondria, the peroxidase isoforms, such as mitAPX, glutathione peroxidase (GPX; EC 1.11.1.9) and peroxiredoxins (Prx; EC 1.11.1.15) isoforms may have an important role in mitochondrial antioxidant defense. Indeed, the silencing of mitochondrial OsGPX3 impairs H_2_O_2_ homeostasis and root and shoot development (Passaia et al., 2013). 

A comparative study between the genes encoding different APX isoforms in different species of Poaceae and how these genes evolved in this group of plants has not yet been performed. Furthermore, little is known about the function of mitochondrial APX isoforms. Therefore, the present work aims to address these two aspects in the understanding of this enzyme family of the plant antioxidant system. To determine the diversity and evolutionary history of the APX in Poaceae species, we performed a genome-wide characterization. We also carried out the functional characterization of rice mitAPX isoforms and compared the impact of silencing the different rice APXs on total APX activity and hydrogen peroxide levels. Our analysis revealed 238 *APX* genes in 24 species from Poaceae. In addition, we identified 30 APX-related (*APX-R*) and 34 APX-like (*APX-L*) genes, proteins closely related to APX. The phylogenetic relationship among these genes, as well as the duplication events that contributed to the expansion of these families, were evaluated. Different from other APX isoforms, the emergence of Poaceae mitAPX occurred independently after eudicot and monocot divergence. The analysis of rice plants silenced to different APX isoforms showed that the double silencing of cAPX and chlAPX isoforms led to decreased total APX activity and increased hydrogen peroxide content. On the other hand, the silencing of pAPX isoforms did not alter these parameters. In contrast to the other APX isoforms, the constitutive silencing of the mitAPX isoforms in rice was not viable, indicating that these isoforms are essential for plant development and their silencing is lethal. This study broadens our understanding of the structural and functional core components of the antioxidant defense in Poaceae species.

## Material and Methods

### Retrieval of APX, APX-R and APX-L amino acid sequences

APX, APX-R and APX-L amino acid sequences of *Brachypodium distachyon* (v3.2), *Panicum virgatum* (v5.1), *Setaria italica* (v2.2), *Zea mays* (RefGen_V4), *Sorghum bicolor* (v3.1.1), *Saccharum spontaneum* (v20190103), *Miscanthus sinensis* (v7.1), *Oryza brachyantha* (v1.4), *Brachypodium stacei* (v1.1), *Brachypodium mexicanum* (v1.1), *Brachypodium hybridum* (v1.1), *Brachypodium sylvaticum* (v1.1), *Panicum hallii* (v3.2), *Paspalum vaginatum* (v3.1), *Urochloa fusca* (v1.1), *Setaria viridis* (v2.1), *Pharus latifolius* (v1.1), *Eleusine coracana* (v1.1), *Oropetium thomaeum* (v1.0), *Triticum aestivum* (IWGSC), *Lolium perenne* (v1.4), *Triticum turgidum* (v1.0) and *Cenchrus americanus* (v1.0) were retrieved from Phytozome v12.1.6 and Dicot Plaza 4.5 databases through BLASTp tool using sequences from rice as bait, and a minimum threshold cutoff of e−20. Sequences were checked by reverse BLASTp in NCBI, and Pfam analysis was used to confirm the presence of conserved domains (El-Gebali et al., 2018).

### Phylogenetic and exon-intron analyses

For phylogenetic analysis, amino acid sequences of APX, APX-R and APX-L proteins were aligned using Multiple Sequence Comparison by Log Expectation tool (MUSCLE) (Edgar, 2004). The phylogenetic tree was made using the maximum likelihood method under the best model selection in MEGA 7.1 software (Tamura et al., 2013) with 1000 replicates of bootstrap statistics. The exon-intron structures of the *APX*, *APX-R* and *APX-L* genes from *Oryza sativa*, *Brachypodium distachyon*, *Panicum virgatum*, *Setaria italica*, *Zea mays*, *Sorghum bicolor* and *Saccharum spontaneum* were examined using the online Gene Structure Display Server (GSDS: http://gsds.cbi.pku.edu.ch) (Guo et al., 2007).

### Calculation of Ka/Ks and divergence time

The nucleotide and amino acid sequences of duplicated gene pairs were aligned and were estimated the number of non-synonymous substitutions per non-synonymous site (Ka), synonymous substitutions per synonymous site (Ks) and Ka/Ks ratio using KaKs_Calculator 2.0 software (Wang et al., 2010). The divergence time between the duplicated genes was calculated through the formula T=Ks/2r, where T represents the divergence time and r represents divergence rate. The divergence rate for monocots was previously presumed to be 6.5 x 10^-9^ (Gaut et al., 1996)

### Structural analysis of APX, APX-R and APX-L proteins

The molecular weight (MW), isoelectric point (pI) and GRAVY (grand average of hydropathy) of the APX, APX-R and APX-L proteins were investigated using the the ProtParam tool (Gasteiger et al., 2005). The conserved motifs in amino acid sequences were analyzed using MEME (Multiple Em for Motif Elicitation) software (http://meme-suite.org/) using the following parameters: number of motifs 1-15 and motif width of 5-50 (Bailey et al., 2009). Prediction of three-dimensional models was performed by AlphaFold software (Jumper et al., 2021), and visualized in Chimera UCSF software. To compare the primary sequence among *Oryza sativa*, *Brachypodium distachyon*, *Panicum virgatum*, *Setaria italica*, *Zea mays*, *Sorghum bicolor* and *Saccharum spontaneum* APX proteins, the translated sequences from their coding regions were aligned with Clustal Omega and analyzed by boxshade interface.

### Prediction of potential *cis-*regulatory elements

The upstream genomic sequences (1000 bp upstream from the translation start codon) of candidate genes were retrieved, and the presence of *cis*-regulatory elements was identified by Plant Promoter Analysis Navigator from the PlantPAN 3.0 database (Chow et al., 2019).

### Plant material and growth conditions

Rice (Oryza sativa L. japonica cv. Nipponbare) seeds were germinated in MS medium (Sigma-Aldrich) at 150 μmol.m^−2^.s^−1^ photosynthetic photon flux density (PPFD), 25 °C, 80% relative humidity and a 12 h photoperiod) One week after being sown, the rice seedlings were transferred to hydroponic growth in 200-mL plastic cups (three seedlings per cup) filled with Hoagland-Arnon’s nutritive solution (Hoagland and Arnon, 1950).

### Quantitative PCR (RT-qPCR)

Real-time PCR experiments were carried out using cDNA synthesized from total RNA purified with TRIzol (Invitrogen®). The samples were treated with DNAase (Invitrogen®) to remove the eventual genomic DNA contamination and complementary DNA (cDNA) was obtained using the SuperscriptTMII (Life Technologies®) reverse transcriptase system and a 24-polyTV primer (Invitrogen®). After synthesis, cDNAs were diluted 10-100 times in sterile water for use in PCR reactions. All reactions were repeated four times, and expression data analyses were performed after comparative quantification of the amplified products using the 2^-ΔΔCt^ method (Livak and Schmittgen, 2001; Schmittgen and Livak, 2008). RT-qPCR reactions were performed in an Applied Biosystems StepOne plus Real Time PCR system (Applied Biosystems^®^).

### APX enzymatic assays

Shoots (approximately 1g) from non-transformed (NT), RNAi*OsAPX1/2*, RNAi*OsAPX4*, RNAi*OsAPX7/8*, RNAi*OsAPX7* and RNAi*OsAPX8* plants were immersed in liquid nitrogen, finely ground to a powder with a mortar and pestle, and 2 mL of 100 mM K-phosphate buffer, pH 6.8, containing 0.1 mM EDTA, were added to allow protein extraction. After centrifugation at 12,000 × g, 15 min, 4 °C, the soluble protein content of the supernatant was quantified using the method described by Bradford (1976) and, subsequently, used to evaluate APX enzymatic activity. The activity of ascorbate peroxidase (APX) was measured by following the ascorbate oxidation by the decrease in absorbance at 290 nm, as previously described by Koshiba (1993).

### Quantitative measurement of H2O2

Measurements of hydrogen peroxide content were performed by extracting from leaves according to Rao et al. (2000) using Ampliflu Red (Sigma-Aldrich) oxidation (Smith et al., 2004). Fluorescence was monitored using a fluorometer at excitation and emission wavelengths of 563 nm and 587 nm, respectively. Calibration was performed by the addition of known quantities of H_2_O_2_.

### Vector construction and plant transformation

A chimeric gene producing mRNA with a hairpin structure (hpRNA) was constructed based on the sequence of the *OsAPX5*, *OsAPX6* and *OsAPX7* (*LOC_Os12g07830*, *LOC_Os12g07820*, and LOC Os04g35520) genes. The following primer pairs were used to amplify a 204 bp sequence common to OsAPX5 and OsAPX6 (5’ - CATACTCGAGG GAGTTGAGTTAG-3’ and 5’ - CTATACTAGTAGGTG GGCATTCT-3’) and a 220 bp fragment from the OsAPX7 (5’ - CTCCGAGCAATCTGGGTGCAAAAAT-3’ and 5’ - GGTACCTCGAGGACTCGTGGTCAGGAAAAGC-3’). PCR product was cloned into the Gateway vector pANDA, in which hairpin RNA is driven by the maize ubiquitin promoter and an intron placed upstream of the inverted repeats (Miki and Shimamoto, 2004). The construct was denominated RNAiOsAPX5/6. Agrobacterium tumefaciens-mediat ed transformation was performed as described previously (Upadhyaya et al., 2002).

### Analysis of the subcellular location of OsAPX5 and OsAPX6 proteins in rice protoplast

The subcellular localization of OsAPX5 and OsAPX6 proteins was experimentally determined in rice protoplasts. The translational fusion of OsAPX5 and OsAPX6 with YFP protein was driven by the CaMV 35S promoter. Protoplast isolation was performed as described by Chen et al. (2006) and protoplast transformation as described by Tao et al. (2002). After transformation, protoplasts were incubated for 24-48 h in the dark at 28 °C before imaging. Fluorescence was monitored using an Olympus FluoView 1000 confocal laser scanning microscope (Olympus, Japan) equipped with a set of filters capable of distinguishing between green and yellow fluorescent protein (GFP and YFP, respectively) and plastid autofluorescence. The images were captured with a high-sensitivity photomultiplier tube detector.

### Statistical analysis

Data were plotted with GRAPHPAD PRISM 5.0 (GraphPad Software Inc., La Jolla, CA, USA) and analyzed by one-way ANOVA and a posteriori Tukey’s test. P-values of 0.05 were considered statistically significant.

## Results

### Identification and phylogenetic analysis of APX family in Poaceae species

**Figure 1 -:**
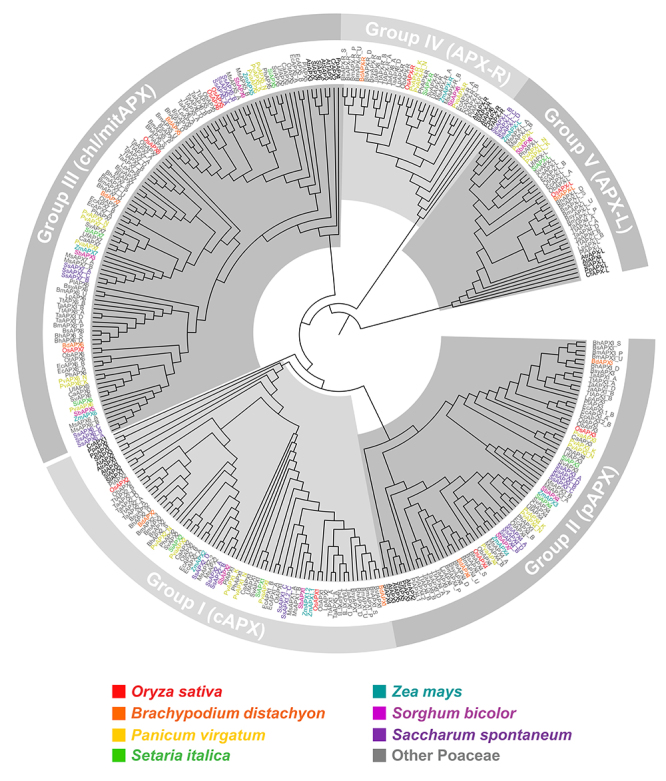
Phylogenetic analysis of APX, APX-R and APX-L proteins. The phylogenetic relationship between APX, APX-R, APX-L was reconstructed using the maximal-likelihood method under the best model selection in IQ-TREE software with 1000 replicates of rapid bootstrap and aLRT statistics. A total of 334 protein sequences were included in the analysis, and ambiguous positions were removed from the alignment. The protein sequences separated in five well-supported clusters: Group I - cytosolic APX (cAPX); Group II - peroxisomal APX (pAPX); Group IIII - mitochondrial/chlroplastic APX (mit/chlAPX); Group IV - APX-related (APX-R); Group V - APX-Like (APX-L). The posterior probabilities are discriminated above each branch.

The use of the OsAPX, OsAPX-R, and OsAPX-L genes as bait against the genome of 24 species from the Poacea family, distributed into 16 genera, allowed us to identify 238 *APX*, 30 *APX-R,* and 34 *APX-L* genes. Phylogenetic analyses revealed a clear divergence among these APX, APX-R, and APX-L genes (Figure 1). Among the APX sequences, there are two main phylogenetic groups resultant of a first dichotomous branching. One branch contains sequences from cAPX and pAPX isoforms, and another one includes the mitAPX and chlAPX sequences. 

The analysis of cAPX sequences, here named as group I, revealed a subsequent and specific duplication event that resulted in two branches of cAPX, named as groups Ia and Ib. Except for *Lolium perenne* and *Urochloa fusca*, which have only one cAPX gene, all analyzed species showed at least one cAPX gene from each phylogenetic subgroups Ia and Ib. Similarly, two branches of pAPX were also observed, here named groups IIa and IIb. These groups are possibly due to a duplication event of an ancestral pAPX. In all analyzed species, pAPX sequences belonging to both groups were found. The sequences of mitAPX, sAPX, and tAPX isoforms are grouped in individual branches, named groups IIIa, IIIb, and IIIc, respectively. In all species, members of each group are present. Among the groups IIIa, IIIb, and IIIc, mitAPX appears to be the more divergent, whereas sAPX and tAPX are possibly resultants of more recent duplication and neofunctionalization events. Here, the APX-R group is named group IV and APX-L as group V. In contrast to *APX* genes, typically only one *APX-R* gene is present in most of the plant species, being absent in *Cenchrus americanus* and *Saccharum spontaneum* genomes.

The APX, APX-R, and APX-L genes identified in Poacea species classified into each phylogenetic group are indicated in Table 1. The genes were named following the rice APX nomenclature, considering the phylogenetic subgroups. Because *APX* genes from *Zea mays* (Liu et al., 2012), *Sorghum bicolor* (Akbudak et al., 2018), and *Triticum aestivum* (Tyagi et al., 2020) were identified previously, we kept the names earlier indicated. Among the analyzed species, *Panicum virgatum*, *Saccharum spontaneum*, *Miscanthus sinensis*, *Brachypodium mexicanum*, *Brachypodium hybridum*, *Eleusine coracana*, *Triticum aestivum*, and *Triticum turgidum* have multiples genomes. The gene haplotypes were named indicating their respective subgenome: *Panicum virgatum* (K and N), *Saccharum spontaneum* (A, B, C and D), *Miscanthus sinensis* (A and B), *Brachypodium mexicanum* (P and U), *Brachypodium hybridum* (D and S), *Eleusine coracana* (A and B), *Triticum aestivum* (A, B and D), and *Triticum turgidum* (A and B) (Table 1). The *APX2* genes from *Brachypodium mexicanum* (*Brame.U002700* and *Brame.U006600*) were found in scaffolds; consequently, we could not identify the subgenomes in these haplotypes. 

**Table 1-:** List of APX, APX-R and APX-L genes identified in Poaceae species classified into phylogenetic groups.

Species name		Genes identified									Total
		Ia	Ib	IIa	IIb	IIIa	IIIb	IIIc	IV	V	
*Brachypodium distachyon*		1	1	1	1	1	1	1	1	1	9
*Brachypodium stacei*		1	1	1	1	1	1	1	1	1	9
*Brachypodium sylvaticum*		1	1	1	1	1	1	1	1	1	9
*Cenchrus americanus*		1	1	1	1	1	1	1	-	1	8
*Lolium perenne*		-	1	1	1	1	1	1	1	1	8
*Oropetium thomaeum*		1	1	1	1	1	1	1	1	1	9
*Oryza brachyantha*		1	1	1	1	1	1	1	1	1	9
*Oryza sativa*		1	1	1	1	2	1	1	1	1	10
*Panicum hallii*		1	1	1	1	1	1	1	1	1	9
*Paspalum vaginatum*		1	1	1	1	1	1	1	1	1	9
*Pharus latifolius*		1	1	1	2	1	1	1	1	1	10
*Setaria italica*		1	1	1	1	1	1	1	1	1	9
*Setaria viridis*		1	1	1	1	1	1	1	1	1	9
*Sorghum bicolor*		1	1	1	1	1	1	1	1	1	9
*Urochloa fusca*		1	-	1	1	1	1	1	1	1	8
*Zea mays*		2	1	1	1	1	1	1	1	1	10
*Brachypodium hybridum*	D	1	1	1	1	1	1	1	1	1	9
	S	1	1	1	1	1	1	1	1	1	9
*Brachypodium mexicanum*	P	1	1	1	1	1	1	1	1	1	9
	U	1	1	1	1	1	1	1	1	1	9
*Eleusine coracana*	A	1	1	1	-	1	1	1	1	1	8
	B	1	1	2	1	1	1	1	1	1	10
*Miscanthus sinensis*	A	1	1	1	1	1	1	1	1	1	9
	B	1	1	1	1	1	1	1	1	1	9
*Panicum virgatum*	K	1	-	1	1	1	1	1	1	1	8
	N	1	1	1	1	1	1	1	1	1	9
*Triticum turgidum*	A	1	1	1	1	1	1	1	1	1	9
	B	1	1	1	1	1	1	1	1	1	9
*Triticum aestivum*	A	1	1	1	1	1	1	1	1	1	9
	B	1	1	1	1	1	1	1	1	1	9
	D	1	1	1	1	1	1	1	1	1	9
*Saccharum spontaneum*	A	-	1	1	1	1	1	1	-	-	6
	B	-	1	1	1	1	1	1	-	1	7
	C	2	-	1	-	1	1	1	-	1	7
	D	-	1	1	1	-	-	-	-	1	4
Total		33	32	36	34	35	34	34	30	34	302

### Structural organization of APX, APX-R, and APX-L genes in Poacea species

**Figure 2-:**
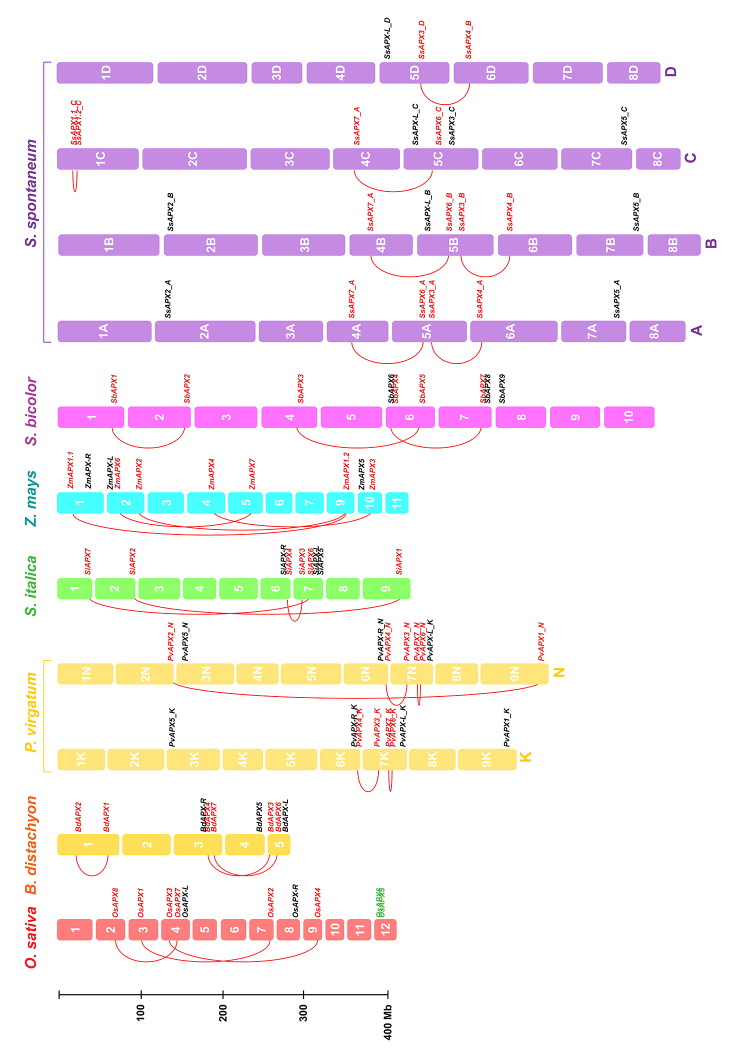
Chromosomal distribution of APX, APX-R and APX-L genes in *Oryza sativa, Brachypodium distachyion, Panicum virgatum* (subgenomes K and N), *Setaria italica*, *Zea mays*, *Sorghum bicolor* and *Saccharum* *spontaneum* (subgenomes A, B, C and D). Chromosome numbers are displayed next to each bar. Red lines indicate segmental duplications and gene duplicated in tandem are indicated in green.

**Figure 3-:**
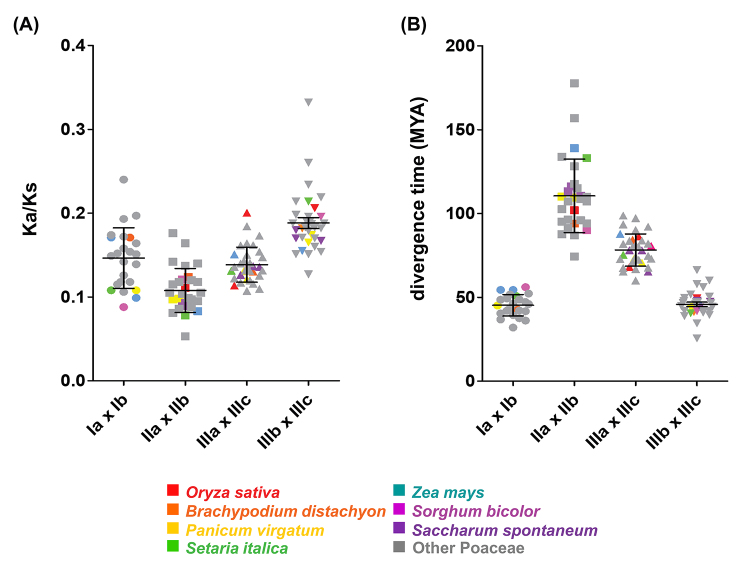
Ratios of non-synonymous to synonymous substitutions (Ka/Ks) and estimated divergence time in APX genes from Poaceae species. **(A)** Ka/Ks ratios of intraspecific duplicated gene pairs from group I (group Ia x group Ib), group II (group IIa x group IIb) and group III (group IIIa and IIIc; group IIIb and IIIc); **(B)** estimated divergence time of duplicated gene pairs from group I, group II and group III. These parameters are determined for 24 Poaceae species and the values for *Oryza sativa*, *Brachypodium distachyon*, *Panicum virgatum*, *Setaria italica*, *Zea mays*, *Sorghum bicolor* and *Saccharum spontaneum* are indicated in colored symbols.

To investigate the relationships among the different genes encoding the APX, APX-R, and APX-L isoforms in Poaceae species, we compared their chromosomal locations and structural organization, among *Oryza sativa*, *Brachypodium distachyon*, *Panicum virgatum*, *Setaria italica*, *Zea mays*, *Sorghum bicolor*, and *Saccharum spontaneum* orthologs. The chromosomal location of *APX*, *APX-R,* and *APX-L* genes in these species, the paralogous genes resultant from duplication events, and the collinearity among the orthologs from the species indicate a close evolutionary relationship among the *APX*, *APX-R,* and *APX-L* orthologous genes of Poaceae species and a variable distribution on different chromosomes (Figure 2, Figure S1). The study of paralogous genes indicates that the pairs of *cAPX*, *pAPX,* and *chlAPX* genes in each species are due to segmental duplication events, whereas the *mitAPX* genes from *Oryza sativa* (*OsAPX5* and *OsAPX6*) are the unique duplicated *APX* gene pair resultant of an *in tandem* duplication event. The Ka/Ks ratios of each duplicated gene pair were <0.1, suggesting the occurrence of purifying selection (Figure 3A).

Our analysis also indicates that the duplication events possibly occurred at different times (Figure 3B; Table 2). Among the different paralogous genes, the duplication of pAPX (groups IIa and IIb) appears to be more ancestral, around 110 million years ago (MYA). Posteriorly, mitAPX (IIIa) branched off from chlAPX approximately 80 MYA. Finally, the duplication events of cAPX (groups Ia and Ib) and chlAPX (groups IIIb and IIIc) are more recent, possibly occurring around 45 MYA. Additionally, in some species, specific duplication events occurred. The *in tandem* duplication of *Oryza sativa* mitAPX may have occurred around 16 MYA. In *Zea mays* and *Saccharum spontaneum*, segmental duplications of cAPX from group Ia probably occurred around 14 and 1 MYA, respectively. As expected, these paralogous genes are very similar, and the physiological importance of these duplication events remains unknown. In APX-R and APX-L groups, duplication events were not observed. It was already demonstrated that gene duplication among APX-R is uncommon, and these genes faced strong negative selection pressure (Dunand et al., 2011; Lazzarotto et al., 2011).

**Table 2-:** Ka/Ks analysis and divergence time between the duplicated APX gene pairs in *Oryza sativa*, *Brachypodium distachyion*, *Panicum virgatum*, *Setaria italica*, *Zea mays*, *Sorghum bicolor* and *Saccharum spontaneum*. Ka. Non-synonymous substitution rate; Ks. Synonymous substitution rate; MYA. Million years ago.

Group	Gene 1	Gene 2	Type	Ka	Ks	Ka/Ks	Date (MYA)
Group I	OsAPX1	OsAPX2	segmental	0.101	0.640	0.158	39.50
	BdAPX1	BdAPX2	segmental	0.121	0.706	0.171	43.59
	PvAPX1_N	PvAPX2_N	segmental	0.079	0.731	0.108	45.13
	SiAPX1	SiAPX2	segmental	0.089	0.823	0.108	50.82
	ZmAPX1.1	ZmAPX2	segmental	0.088	0.882	0.099	54.46
	ZmAPX1.2	ZmAPX2	segmental	0.151	0.882	0.171	54.45
	SbAPX1	SbAPX2	segmental	0.080	0.909	0.088	56.12
	ZmAPX1.1	ZmAPX1.2	segmental	0.011	0.228	0.047	14.06
	SsAPX1.1_C	SsAPX1.2_C	segmental	0.002	0.017	0.104	1.04
Group II	OsAPX3	OsAPX4	segmental	0.183	1.652	0.111	101.97
	BdAPX3	BdAPX4	segmental	0.189	1.523	0.124	94.01
	PvAPX3_K	PvAPX4_K	segmental	0.175	1.784	0.098	110.12
	PvAPX3_N	PvAPX4_N	segmental	0.172	1.774	0.097	109.50
	SiAPX3	SiAPX4	segmental	0.168	2.156	0.078	133.08
	ZmAPX3	ZmAPX4	segmental	0.187	2.251	0.083	138.93
	SbAPX4	SbAPX7	segmental	0.177	1.462	0.121	90.24
	SsAPX3_A	SsAPX4_A	segmental	0.166	1.790	0.093	110.52
	SsAPX3_B	SsAPX4_B	segmental	0.164	1.835	0.089	113.27
	SsAPX3_D	SsAPX4_D	segmental	0.168	1.882	0.089	116.19
Group III	OsAPX5	OsAPX6	tandem	0.057	0.275	0.208	16.95
	OsAPX7	OsAPX8	segmental	0.165	0.800	0.206	49.41
	BdAPX6	BdAPX7	segmental	0.122	0.668	0.182	41.26
	PvAPX6_K	PvAPX7_K	segmental	0.118	0.718	0.165	44.31
	PvAPX6_N	PvAPX7_N	segmental	0.124	0.711	0.174	43.86
	SiAPX6	SiAPX7	segmental	0.141	0.658	0.214	40.60
	ZmAPX6	ZmAPX7	segmental	0.113	0.731	0.155	45.10
	SbAPX5	SbAPX3	segmental	0.133	0.678	0.196	41.87
	SsAPX6_A	SsAPX7_A	segmental	0.128	0.715	0.180	44.12
	SsAPX6_B	SsAPX7_B	segmental	0.132	0.777	0.170	47.96
	SsAPX6_C	SsAPX7_C	segmental	0.129	0.769	0.167	47.49

**Figure 4-:**
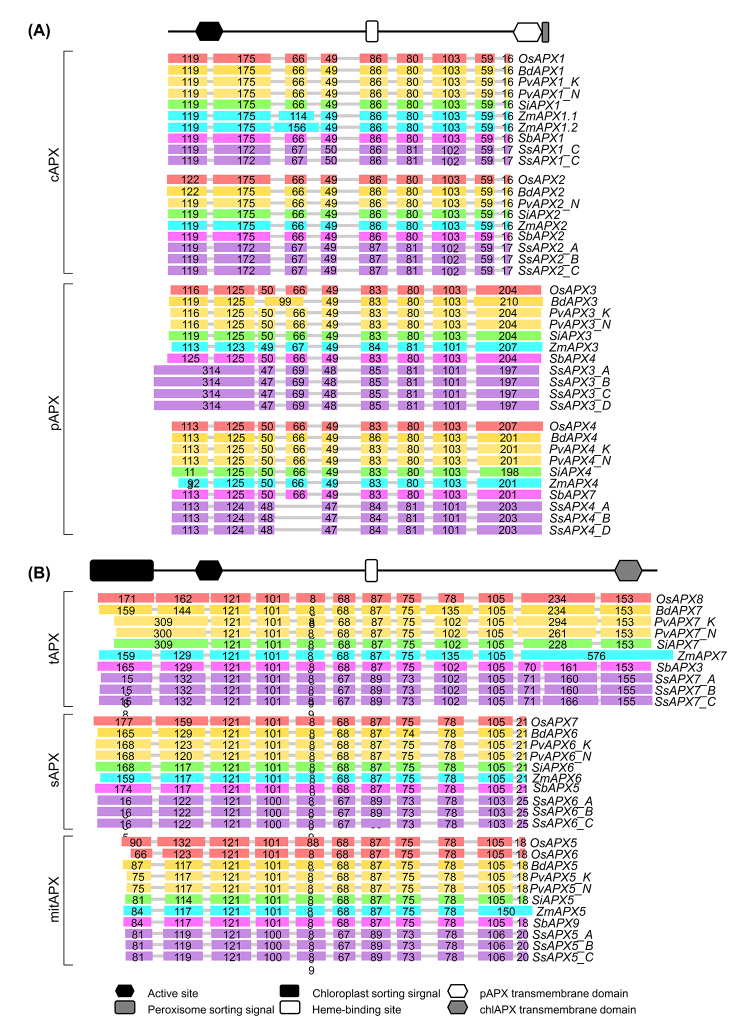
Exon-intron structure APX genes from *Oryza sativa*, *Brachypodium distachyion*, *Panicum virgatum*, *Setaria italica*, *Zea mays*, *Sorghum bicolor* and *Saccharum spontaneum* **(A)** cAPX and pAPX, and **(B)** tAPX, sAPX and mitAPX. For all genes, grey lines represent introns and the lengths of exons are exhibited proportionally.

The analysis of the structural organization of *APX*, *APX-R,* and *APX-L* genes reveals a high degree of conservation in exon-intron structure among the sequences belonging to the same phylogenetic group (Figure 4). The cAPX and pAPX subfamilies show a similar exon-intron structure, except for exon 2 from *cAPX* and the last exon from *pAPX* genes. The *cAPX* exon 2 is equivalent to exons 2 and 3 from *pAPX*, and the last exon of *pAPX* genes encodes the *pAPX* transmembrane domain and the peroxisome sorting signal, which is absent in *cAPX* genes (Figure 4A). In both groups, the APX active site is encoded by exons 1 and 2, and the heme-binding site is encoded by exon 5 from *cAPX* and exon 6 from *pAPX* genes. Although the exon-intron structure is highly conservated among different species, *BdAPX3* has a longer exon 3, which is most likely due to a fusion of equivalents to exon 3 and 4 from other species. In addition, the equivalent to exon 4 from the *SsAPX4* gene appears to be not present.

The exon-intron structure is also highly conserved among *tAPX*, *sAPX,* and *mitAPX* genes (Figure 4B). In these groups, the chloroplast and mitochondria sorting signals are found in exon 1, the APX active site is encoded by exons 2 and 3, and the heme-binding site is encoded by exon 7. *tAPX* genes have a longer exon 11 and an additional exon 12, which contain the coding sequence to the chlAPX transmembrane domain, responsible for anchoring tAPX in the thylakoid membrane. Despite this general similarity, some singularities were observed. Exon 1 from *PvAPX7_K*, *PvAPX7_K*, and *SiAPX7* appears to be a fusion of the equivalents to exons 1 and 2 from other species. In *ZmAPX7*, the equivalents to exons 11 and 12 appear to be fused as a unique and longer exon 11. On the other hand, in *SbAPX3*, *SsAPX7_A*, *SsAPX7_B*, and *SsAPX7_C*, exon 11 is divided into two exons. The exon-intron structure of the *APX-R* and *APX-L* genes displays more divergency in comparison to the *APX* genes (Figure S2). The analysis of the putative promoter regions of the Poaceae *APX*, *APX-R,* and *APX-L* genes identified many putative cis-acting elements responsive to phytohormone signaling and environmental stresses (Table S1 - https://1drv.ms/u/s!aihfiliuspreg8ysv9pqxjfrohju8w?e=hrviil, Figure S3).

### Protein sequence analyses of APX, APX-R, and APX-L in Poaceae species

The Poaceae *APX*, *APX-R,* and *APX-L* genes encode polypeptides of 155-1521 amino acid residues, 27.35-98.75 Kda, and 5.75-9.60 PI values (Table S2, https://1drv.ms/u/s!aihfiliuspreg8ysv9pqxjfrohju8w?e=hrviil). The sequence variations are correlated with their respective subfamilies and, in part, can be explained by the presence of transit peptides and transmembrane domains. Among the analyzed APX, sequences from groups IIIa, IIIb, and IIIc displayed the highest instability index. The lability of chlAPX proteins has been previously demonstrated in different species, and high levels of endogenous ascorbic acid are necessary to prevent their inactivation (Shikanai et al., 1998; Miyagawa et al., 2000; Yoshimura et al., 2000; Shigeoka et al., 2002; Liu et al., 2008). Further, APX, APX-R, and APX-L sequences show low GRAVY values, suggesting better interactions with water due to their hydrophilic nature.

**Figure 5-:**
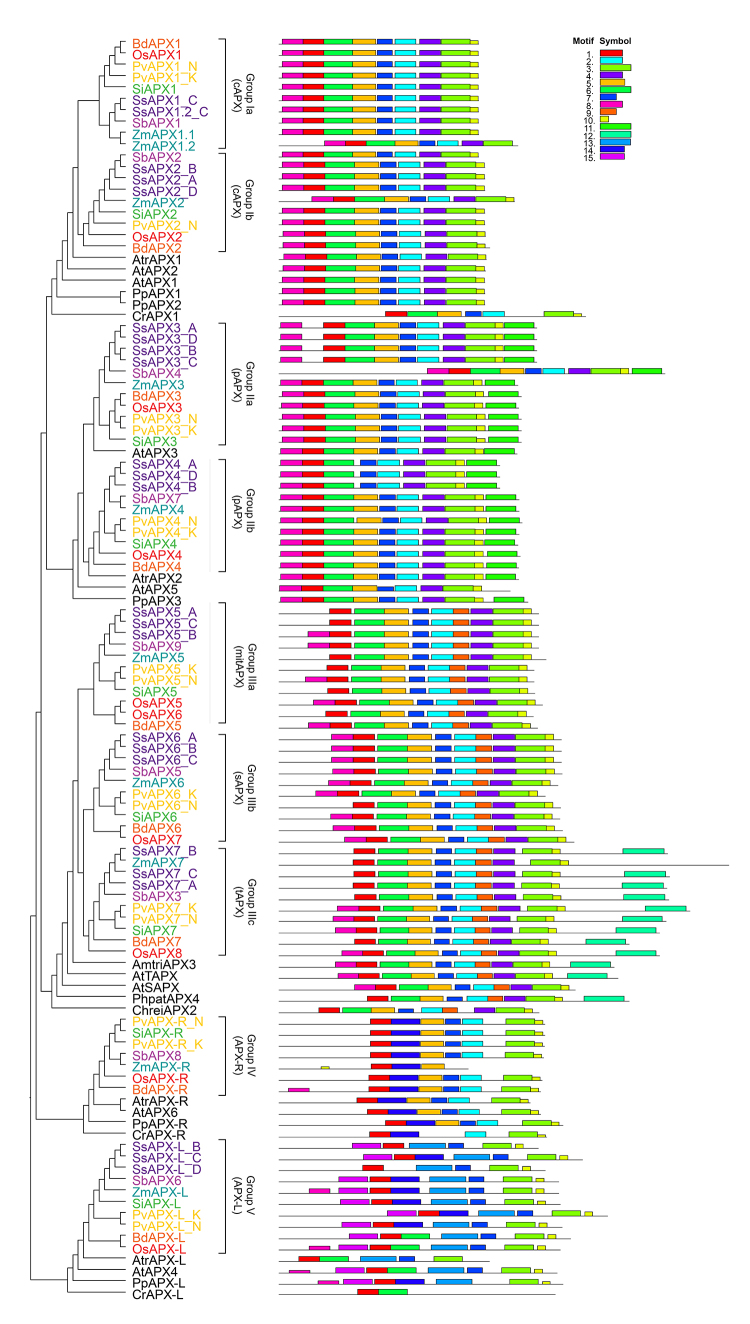
Conserved motifs of APX, APX-R and APX-L proteins from *Chlamydomonas reinhardtii* (Chrei), *Physcomitrella patens* (Phpat), *Amborella trichopoda* (Amtri), *Arabidopsis thaliana* (At), *Oryza sativa* (Os), *Brachypodium distachyion* (Bd), *Panicum virgatum* (Pv), *Setaria italica* (Si), *Zea mays* (Zm), *Sorghum bicolor* (Sb) and *Saccharum spontaneum* (Ss). The phylogenetic relationship between APX, APX-R, APX-L was reconstructed using the maximal-likelihood method under the best model selection in IQ-TREE software with 1000 replicates of rapid bootstrap and aLRT statistics. A total of 111 protein sequences were included in the analysis, and ambiguous positions were removed from the alignment. All 15 conserved motifs in APX, APX-R and APX-L proteins were identified by MEME software and indicated by a colored box. The lines represent the non-conserved sequences, and the length of motifs in each protein is presented proportionally.

To compare the motifs shared within APX, APX-R, and APX-L sequences, the MEME motif search tool was employed. This analysis identified 15 distinct conserved motifs (Figure 5). Among the APX sequences, the cAPX (groups Ia and Ib), pAPX (groups IIa and IIb), mitAPX (group IIIa), and chlAPX (groups IIIb and IIIc) isoforms show almost the same motif composition pattern. Motif 11 is found only in the pAPX subfamily and is equivalent to a peroxisomal targeting signal. In groups IIIa, IIIb, and IIIc, an N-terminal extension was found, which corresponds to the chloroplast/mitochondrial transit peptide. Among these groups, the sequences of group IIIc show a C-terminal extension with the transmembrane domain related to thylakoid membrane anchoring (motif 12). As expected, the APX-R and APX-L sequences (groups IV and V) show a distinct motif composition. The sequence logos for the 15 conserved motifs of APX, APX-R, and APX-L proteins are shown in Figure S4.

**Figure 6-:**
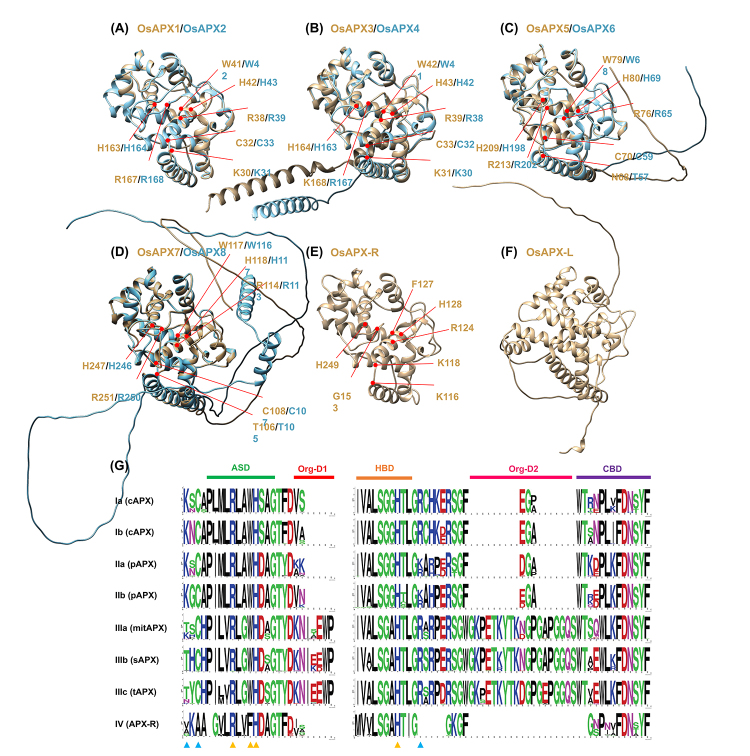
Structure and protein sequence analysis of APX, APX-R and APX-L in Poaceae species. The tertiary structure of OsAPX1 and OsAPX2 (cAPX) **(A)**, OsAPX3 and OsAPX4 (pAPX) **(B)**, OsAPX5 and OsAPX6 (mitAPX) **(C)**, OsAPX7 and OsAPX8 (chlAPX) **(D)**, OsAPX-R **(E)** and OsAPX-L **(F)** was predicted by AlphaFold algorithm. The amino acid residues involved in ascorbate bind and catalytic activity are indicated. **(G)** Multiple sequence alignments of protein sequences. Green, red, orange, pink and purple bars represent the active site domain (ASD), organellar signature domain 1 (Org-D1), heme-binding domain (HBD), organellar signature domain 2 (Org-D2) and cation binding domain (CBD) present in all phylogenetic groups. Blue triangles represent the amino acid residues involved in ascorbate bind residues (Lys, Cys and Arg) and yellow triangles represent catalytic residues (Arg, Trp, His, His).

Because APX protein sequences are highly conserved among different species, the protein structure prediction was performed for the rice APX, APX-R, and APX-L sequences, using Swiss-Model software to construct three-dimensional models. Figure 6 shows the structural models of *Oryza sativa* OsAPX1, OsAPX2, OsAPX3, OsAPX4, OsAPX5, OsAPX6, OsAPX7, OsAPX8, OsAPX-R, and OsAPX-L proteins with the indication of amino acids residues related to catalytic activity and ascorbate binding. As expected, the APX models show high structural conservation with similar helices and strands (Figures 6A-D). On the other hand, the APX-R and APX-L structural models are more divergent (Figures 6E , F). We also analyzed APX, APX-R, and APX-L amino acid sequences, comparing *Oryza sativa*, *Brachypodium distachyon*, *Panicum virgatum*, *Setaria italica*, *Zea mays*, *Sorghum bicolor* and *Saccharum spontaneum* orthologs. The alignments of cAPX, pAPX, mitAPX, sAPX, tAPX, APX-R, and APX-L amino acid sequences are indicated in Figures S5, S6, S7, S8, S9, S10, and S11. The sequence logos for the active site, heme-binding, cation-binding organellar signature domains of cAPX, pAPX, mitAPX, sAPX, tAPX, and APX-R are provided in Figure 6G. Our analysis showed that the amino acid residues related to catalytic activity and ascorbate binding, as well as the active site, heme-binding, and cation-binding domains, are conserved in cAPX, pAPX, mitAPX, sAPX, and tAPX sequences. The analysis of APX-R and APX-L demonstrated several divergences in comparison to APX. Despite the divergent motif composition and the low conservation of the APX active site, the APX-R protein shows high conservation in the heme-binding site and the presence of all amino acid residues described as essential to peroxidase activity (Figures 6E, G, and S10). On the other hand, APX-L sequences demonstrated distinct motif patterns, low conservation in the heme-binding and active sites, as well as the absence of catalytic residues described to APX or other enzymes with peroxidase activity (Figures 6F and S11). 

### Comparative analysis of rice plants silenced to *OsAPX1*, *OsAPX2*, *OsAPX3*, *OsAPX4*, *OsAPX7*, and *OsAPX8* genes

Plants silenced for the different rice APX genes were previously obtained. This knock-down collection represents an important tool for functional analysis that is not available for other species. The phenotypic effect of the knockdown of each of these genes differs significantly (Rosa et al., 2010; Caverzan et al., 2014; Souza *et al.*, 2015; Ribeiro et al., 2017; Jardim-Messeder et al., 2018). However, to date, no comparative analysis has been performed with all of these plants. Here, in addition to the analysis of RNAi plants previously generated, namely RNAi*OsAPX1/2* (Rosa *et al.*, 2010), RNAi*OsAPX4* (Ribeiro *et al.*, 2017), RNAi*OsAPX7/8* (Caverzan *et al.*, 2014), and RNAi*OsAPX8* (Jardim-Messeder *et al.*, 2018), we attempted to produce knockdown plants for the mitochondrial isoforms *OsAPX5, OsAPX6,* and *OsAPX7* individually (single mutants). 

Due to the high similarity among *OsAPX5* and *OsAPX6* genes, it was not possible to design individual RNAi for single silencing. Hence, the RNAi was projected to silence both genes simultaneously. After several rounds of transformation experiments, we were not able to obtain embryogenic callus transformed with RNAi*OsAPX5/6* construction, indicating that the double knockdown is lethal and leads to Calli necrosis. On the other hand, we generated different lines single silenced to the *OsAPX7* gene. Two independent lines of RNAi*OsAPX7* plants, named “line a” and “line b”, were analyzed.

**Figure 7-:**
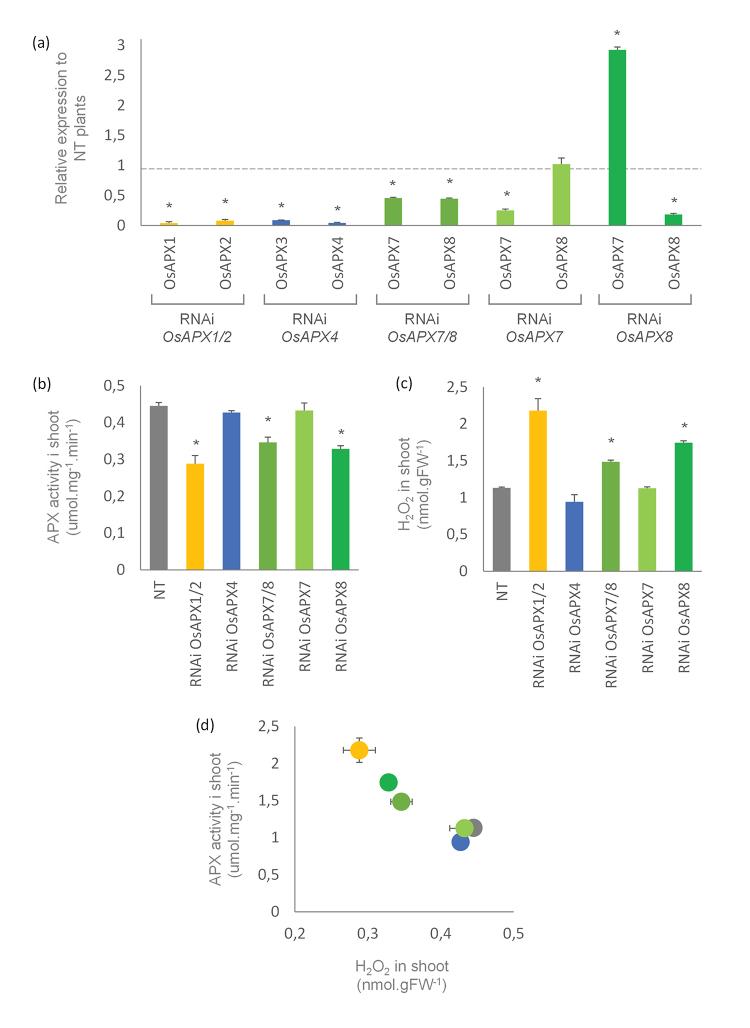
APX activity and hydrogen peroxide content in shoots from rice plants silenced to cAPX, pAPX and chlAPX isoforms. **(A)** Quantitative determination of APX expression in shoots from RNAi OsAPX1/2, RNAi OsAPX4, RNAi OsAPX7/8, RNAi OsAPX7 and RNAi OsAPX8 plants. The values are expressed relatively to NT plants. **(B)** Measurement of APX activity in shoots from NT and silenced plants. **(C)** Hydrogen peroxide content in shoots from NT and silenced plants. **(D)** The relationship among the APX activity and hydrogen peroxide content in shoots from all plants analyzed. The values represent the media ± SE of at least three independent experiments.

The RT-qPCR analysis confirmed the knockdown of the selected genes, with different levels of reduction: 96% for *APX1* and 92% for *APX*2 in RNAi*OsAPX1/2; 91%* to *APX3* and *96%* to *APX4* in RNAi*OsAPX4; 55% to* APX7 and APX8 in RNAi*OsAPX7/8; 82% to APX8* in RNAiOsAPX8 plants*.* In the single-silenced RNAi*OsAPX7* plants, the *OsAPX7* transcript was reduced by 71% and 76% in lines a and b, respectively. In both lines, the *OsAPX8* transcript levels were not altered, confirming that only *OsAPX7* was silenced in these plants (Figure 7A).

The biochemical analysis of the shoot of transformed plants shows that the double silencing of *OsAPX1, OsAPX2*, *OsAPX7,* and *OsAPX8*, and the individual silencing of OsAPX8 decreased total APX activity. In RNAi*OsAPX1/2* plants, the APX activity was reduced by approximately 35%, whereas in RNAi*OsAPX7/8* and RNAi*OsAPX8* plants, the reduction was 22% and 26%, respectively. On the other hand, APX activities in RNAi*OsAPX4* and RNAi*OsAPX7* plants were not altered (Figure 7B). In all analyzed plants, the reduction of APX activity was accompanied by an increase in hydrogen peroxide levels. The RNAi*OsAPX1/2* plants showed the highest hydrogen peroxide levels, by approximately 2 folds compared to NT plants. In RNAi*OsAPX7/8* and RNAi*OsAPX8* plants, the hydrogen peroxide levels were increased by about 31% and 54%, respectively, but no changes were found in RNAi*OsAPX4* and RNAi*OsAPX7* plants (Figure 7C). These results demonstrated that the reduction of APX activity is directly related to increased hydrogen peroxide in shoots from rice plants (Figure 7D), despite that, we should not discard a compensatory mechanism involving other antioxidant enzymes in response to the alterations in the APX expression levels. These plants were previously evaluated phenotypically (Rosa et al., 2010; Caverzan et al., 2014; Souza *et al.*, 2015; Ribeiro et al., 2017; Jardim-Messeder et al., 2018). The relationship of these results with the phenotypic data will be discussed later. 

### Characterization of mitochondrial APX isoforms in rice

**Figure 8 -:**
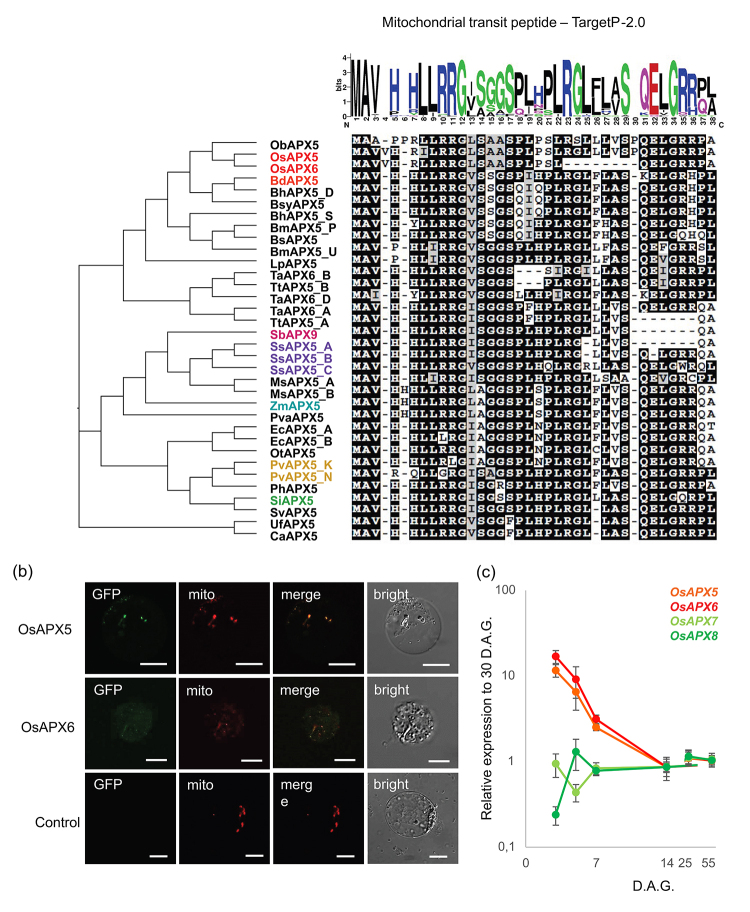
Group IIIa APX are specific to Poacea specie and located in mitochondria. **(A)** Deduced amino acid alignment of predicted mitochondrial transit peptide of group IIIa APX from Poacea species. The sequences were aligned by Clustal Omega and the conserved amino acids are labeled in black. The phylogenetic relationship between APX, APX-R, APX-L was reconstructed using the maximal-likelihood method under the best model selection in IQ-TREE software with 1000 replicates of rapid bootstrap and aLRT statistics. The logos were identified by MEME software. The character and size of each logo represent the proportion of an amino acid at the specific site. **(B)** Subcellular localization of the OsAPX5 and OsAPX6 protein in rice etiolated protoplasts throught transient expression of the 35S-OsAPX5::YFP and 35S-OsAPX6::YFP. Green signals indicate YFP fluorescence; red signals indicate mitochondria location by MitoTracker fluorescence and yellow signals is the merged image. The negative control of YFP fluorescence is indicated. **(C)** Quantitative determination of OsAPX5 (orange), OsAPX6 (red), OsAPX7 (light green) and OsAPX8 (dark green) genes by RT-qPCR in shoot during rice development relative to 60 days after germination (D.A.G.) The values were normalized by at least three constitutive genes and represent the media ± SE of at least three independent experiments.

In each Poaceae genome analyzed in this work, at least one predicted mitAPX isoform was found. The exception was rice, which has two APX isoforms generated by a recent *in tandem* duplication (Figure 2; Table 2). The alignment of the predicted N-terminal mitochondrial transit peptide is shown in Figure 8A, indicating a high conservation among the orthologous of mitAPX. This analysis revealed the presence of positively charged residues and at least one amphipathic α-helix, important for the import of proteins into mitochondria but not chloroplasts (Ge et al., 2014). 

The subcellular localization of OsAPX5 and OsAPX6 were analyzed by YFP fusion in rice protoplasts. Confocal analysis of protoplasts expressing 35S-OsAPX5::YFP and 35S-OsAPX6::YFP fusions revealed that OsAPX5 and OsAPX6 were localized exclusively in mitochondria (Figure 8B). 

We also compared the expression pattern of *mitAPX* with *chlAPX* isoforms during the initial development. Our results show that the expression patterns of the *OsAPX5* and *OsAPX6* genes are highly similar (Figure 8C), with substantial expression in the early stages and then decreasing throughout development. In 3-day-old plants, the expression of *OsAPX5* and *OsAPX6* genes was at least 10-20 folds higher compared with 2-month-old plants. These results suggest that the *mitAPX* isoforms can play an important role during the initial development, potentially explaining the non-viability of embryonic calli transformed with the RNAiOsAPX5/6 construction. Different from mitAPX isoforms, chlAPXs show a different pattern of expression. *OsAPX8* appears to increase during development, whereas the expression of OsAPX7 is more constant.

## Discussion

In the present study, we demonstrated a high analogy in the number and high evolutionary conservation in *APX*, *APX-R,* and *APX-L* in different Poaceae species, with seven *APX*, one *APX-R,* and one *APX-L* gene in most of the diploid plants analyzed. In tetraploid genomes, such as *Brachypodium hybridum*, *Brachypodium mexicanum*, *Miscanthus sinensis*, and *Triticum turgidum*, twice the number of genes were identified, with 14 *APX*, two *APX-R,* and two *APX-L*. In *Triticum aestivum*, which has a hexaploid nature, three times the number of *APX*, *APX-R,* and *APX-L* genes were observed, whereas in *Saccharum spontaneum*, which has an octaploid genome, the number of identified genes was almost quadruple. 

In addition to the ploidy level of each species analyzed, the expansion of APX families occurred due to gene duplication events, which are important for genetic diversity. The phylogenetic analysis showed two main branches of APX sequences, confirming the hypothesis that *APX* isoforms diverged through a duplication event in an ancestral *APX*, generating the non-organellar and organellar isoforms (Teixeira et al., 2004). Later, other duplications and neofunctionalization events allowed the divergence of *cAPX* and *pAPX* in one branch and the divergence of mitAPX and chlAPX in the second branch. As suggested by Lazzarotto et al. (2021a) and the localization of APX-R and APX-L sequences as basal groups, the divergence of *APX*, *APX-R,* and *APX-L* genes possibly occurred before the APX isoform specializations.

More recent duplication and neofunctionalization events in APX genes have allowed the emergence of at least two cAPX and pAPX isoforms and new *chlAPX* genes encoding proteins exclusively soluble in the stroma (sAPX) or bound to the thylakoid membrane (tAPX). The phylogenetic tree indicates that this duplication event occurred exclusively in Poaceae species. In addition, it has been proposed that monocots branched from eudicots at least 140-150 MYA in the late Jurassic-early Cretaceous period (Chaw et al., 2004) and, thus, before the proposed divergence time of *cAPX*, *pAPX*, and *chlAPX* genes (45, 110, and 46 MYA, respectively). Based on these data and the phylogenetic analysis, we propose that these duplication events of APX paralogous genes are specific to monocot species. Despite different duplication events in APX sequences, only one *APX-R* and *APX-L* gene were found in all of the analyzed species. Indeed, previous works demonstrated that APX-R duplication is not acceptable, being attributed to gene loss during evolution (Lazzarotto et al., 2011, 2015).

The intron-exon organization of the *APX*, *APX-R,* and *APX-L* genes from Poaceae species is highly similar to that previously verified in eudicots, such as *Arabidopsis thaliana* (Ozyigit et al., 2016) and *Gossypium hirsutum* (Tao et al., 2018), suggesting a conserved gene architecture in higher plants, including monocots and eudicots species.

The APX structure and catalytic mechanism have been extensively studied, demonstrating the existence of two typical domains of heme peroxidases: the active site and the heme-binding site (Patterson and Poulos, 1995; Mandelman et al., 1998; Raven, 2003; Sharp et al., 2003). These sites contain two pivotal histidine residues essential for APX activity, referred to as proximal and distal histidines. The proximal histidine is involved in heme binding, and the distal is present in the active site functioning in the reaction with hydrogen peroxide (Henrissat et al., 1990). This structure is highly conserved among APX proteins but divergent in APX-R and APX-L, where the active and heme-binding sites are degenerated. These data are consistent with the observation that these proteins do not show ascorbate peroxidase activity (Granlund et al., 2009; Lundberg et al., 2011; Lazzarotto et al., 2021b). 

Despite the divergent motif composition and low conservation of the APX active site, the APX-R proteins show a significant level of conservation in the heme-binding site and the presence of all amino acid residues described as essential for peroxidase activity. These data indicate that APX-R can be a heme peroxidase but may not recognize ascorbate as a substrate, as already demonstrated for the APX-R from arabidopsis (AtAPX6) that can reduce hydrogen peroxide in the presence of pyrogallol and guaiacol, as described for other heme peroxidases, but not in the presence of ascorbate (Lazzarotto et al., 2021b). In addition, the functional characterization of arabidopsis knockout mutants (*apx6-1*) (Chen et al., 2014) and overexpression lines (Lazzarotto *et al.*, 2021b) suggests that APX-R plays an important role in oxidative protection, mainly during seed development and germination. 

Structural and biochemical analysis have showed that while CCP catalysis relies on the formation of a protein-based radical, APX display enzymatic activity through a porphyrin-based radical, exhibiting therefore significant differences in catalytic mechanisms. Sequence analysis indicate that APX-R might also display enzymatic activity through a porphyrin-based radical, similarly to what has been observed for APX (Lazzarotto et al., 2015, 2021b). Despite APX-R real substrate is still unknown, the capacity of APX-R oxidase cytochrome C is unlikely.

The analysis of APX-L sequences shows distinct motif patterns, low conservation in the heme-binding and active sites, as well as the absence of catalytic residues described to APX or other enzymes with peroxidase activity. These data corroborate the hypothesis that APX-L is possibly neither an APX nor a peroxidase (Lazzarotto et al., 2021b). Previous works demonstrated that in arabidopsis, APX-L (AtAPX4, also termed TL29) is a luminal protein associated with photosystem II (PSII) that does not present peroxidase activity (Granlund et al., 2009; Lundberg et al., 2011). Because arabidopsis *apx4* knockout mutants show an increase in hydrogen peroxide accumulation, it has been proposed that the heme group in APX-L proteins scavenges the high-energy electrons derived from photosystem II or the oxygen-evolving complex, acting in the antioxidant defense (Wang et al., 2014). 

Rice plants double silenced to both cAPX genes (*OsAPX1* and *OsAPX2*) exhibited the highest decrease in shoot APX activity and the highest increase in hydrogen peroxide levels among the different transgenic lines silenced for *OsAPX* genes, indicating that these isoforms exert an important role in the control of hydrogen peroxide levels. Nevertheless, previous work has demonstrated that RNAi*OsAPX1/2* plants show a normal phenotype and development (Rosa et al., 2010) and no changes in the responses to salt and osmotic stresses (Cunha et al., 2016). This normal phenotype can be explained by a compensatory mechanism promoted by the altered expression of several genes associated with antioxidant defense and photosynthesis, as well as increased CAT and SOD activities (Ribeiro *et al.*, 2012). 

The analysis of RNAi*OsAPX4* plants, which display both OsAPX4 and OsAPX3 knocked down, do not show changes in the shoot APX activity and hydrogen peroxide content. These plants exhibit an early senescence phenotype compared to NT plants (Souza *et al.*, 2015; Ribeiro et al., 2017). Indeed, in a rice early senescence leaf mutant (*esf*), the early senescence phenotype is associated with the repressed expression of *OsAPX4* and increased hydrogen peroxide production in peroxisomes (Li et al., 2014). 

The analysis found that the knockdown of stromal *OsAPX7* did not impair the total APX activity and plant development. In addition, these plants did not exhibit changes in hydrogen peroxide content. These data contrast with the previous results of the double silencing of OsAPX7 and OsAPX8 in RNAi*OsAPX7/8* and single-silenced OsAPX8 in RNAi*OsAPX8* plants, which show a decrease in shoot APX activity and an increased hydrogen peroxide content. However, these data indicate that the *OsAPX8* exerts the main role in the chloroplast antioxidant defense under normal conditions. Indeed, the hydrogen peroxide reduction by tAPX is considered the first layer of antioxidant defense in chloroplasts, whereas its removal by sAPX in the stroma constitutes a second defense layer (Maruta et al., 2016; Caverzan et al., 2019). As demonstrated by Jardim-Messeder et al. (2018), under stress conditions, *OsAPX7* is induced, whereas *OsAPX8* is repressed. These data indicate that the sAPX and tAPX isoforms can exert differential roles under stress response.

Although the silencing of cAPX, pAPX, and chlAPX isoforms produces viable plants, the regeneration of rice plants from embryogenic callus transformed with RNAi to mitAPX isoforms led to an extensive process of necrosis of calli. These results indicated that the silencing of mitAPX is not possible, and these isoforms may exert a central role in the control of mitochondrial hydrogen peroxide levels during plant development. In addition, these results may be related to the fact that mitochondria are key regulators of programmed cell death in plants and that increasing the level of ROS in mitochondria can lead to programmed cell death (Mittler et al., 2002; Scandalios, 2002). The analysis of subcellular localization of OsAPX5 and OsAPX6 indicates that the mitAPX genes encode proteins targeted exclusively to mitochondria and not to chloroplasts. The alignment of the predicted N-terminal mitochondrial transit peptide in all analyzed mitAPX orthologous genes suggested that this localization is also verified in all investigated Poaceae species. These results are different from those observed in Arabidopsis, in which the sAPX isoform is dual targeted to mitochondria and chloroplasts (Chew et al., 2003; Xu et al., 2013). In*Populus tomentosa*, a dual-targeted APX and a second isoform specifically targeted to mitochondria were also experimentally demonstrated (Yin et al., 2019). Despite mitAPX also being observed in eudicot species, our phylogenetic and duplication analyses demonstrated that the emergence of Poaceae mitAPX occurred independently after eudicot and monocot divergence.

This work reinforces the knowledge regarding the phylogenetic, syntenic, structural, and molecular relationships of *APX*, *APX-R,* and *APX-L* genes from Poaceae species, particularly in rice, leading to a foundation for further functional exploration and possible biotechnological application of *APX* genes. The Poaceae species are responsible for most calories consumed by the world’s population, and compressive analyses of gene families related to ROS metabolism and stress response are essential for understanding how different cultures respond appropriately to environmental stresses.

## Supplementary material

The following online material is available for this article:

Figure S1 - Collinearity among *APX*, *APX-R* and *APX-L* genes in *Oryza sativa*, *Brachypodium distachyion*, *Panicum virgatum* (subgenomes K and N), *Setaria italica*, *Zea mays*, *Sorghum bicolor* and *Saccharum spontaneum* (subgenomes A, B, C and D).

Figure S2 - Exon-intron structure *APX-R* and *APX-L* genes from *Oryza sativa*, *Brachypodium distachyion*, *Panicum virgatum*, *Setaria italica*, *Zea mays*, *Sorghum bicolor* and *Saccharum spontaneum*.

Figure S3 - Organization of *cis*-regulatory elements related to hormone responsiveness and environmental stress in *APX*, *APX-R* and *APX-L* genes from *Oryza sativa*, *Brachypodium distachyon*, *Panicum virgatum*, *Setaria italica*, *Zea mays*, *Sorghum bicolor* and *Saccharum spontaneum*.

Figure S4 - Sequence logos for the conserved motifs of APX, APX-R and APX-L proteins.

Figure S5 - Protein sequence alignment of cytoplasmatic APX (groups Ia and Ib) from *Oryza sativa* (*Os*), *Brachypodium distachyion* (*Bd*), *Panicum virgatum* (*Pv*), *Setaria italica* (*Si*), *Zea mays* (*Zm*), *Sorghum bicolor* (*Sb*) and *Saccharum spontaneum* (*Ss*).

Figure S6 - Protein sequence alignment of peroxisomal APX (groups IIa and IIb) from *Oryza sativa* (*Os*), *Brachypodium distachyion* (*Bd*), *Panicum virgatum* (*Pv*), *Setaria italica* (*Si*), *Zea mays* (*Zm*), *Sorghum bicolor* (*Sb*) and *Saccharum spontaneum* (*Ss*).

Figure S7 - Protein sequence alignment of mitochondrial APX (group IIIa) from *Oryza sativa* (*Os*), *Brachypodium distachyion* (*Bd*), *Panicum virgatum* (*Pv*), *Setaria italica* (*Si*), *Zea mays* (*Zm*), *Sorghum bicolor* (*Sb*) and *Saccharum spontaneum* (*Ss*).

Figure S8 - Protein sequence alignment of stromal APX (group IIIb) from *Oryza sativa* (*Os*), *Brachypodium distachyion* (*Bd*), *Panicum virgatum* (*Pv*), *Setaria italica* (*Si*), *Zea mays* (*Zm*), *Sorghum bicolor* (*Sb*) and *Saccharum spontaneum* (*Ss*).

Figure S9 - Protein sequence alignment of thylakoid APX (group IIIc) from *Oryza sativa* (*Os*), *Brachypodium distachyion* (*Bd*), *Panicum virgatum* (*Pv*), *Setaria italica* (*Si*), *Zea mays* (*Zm*), *Sorghum bicolor* (*Sb*) and *Saccharum spontaneum* (*Ss*).

Figure S10 - Protein sequence alignment of APX-related (group IV) from *Oryza sativa* (*Os*), *Brachypodium distachyion* (*Bd*), *Panicum virgatum* (*Pv*), *Setaria italica* (*Si*), *Zea mays* (*Zm*), *Sorghum bicolor* (*Sb*) and *Saccharum spontaneum* (*Ss*).

Figure S11 - Protein sequence alignment of APX-like (group V) from *Oryza sativa* (*Os*), *Brachypodium distachyion* (*Bd*), *Panicum virgatum* (*Pv*), *Setaria italica* (*Si*), *Zea mays* (*Zm*), *Sorghum bicolor* (*Sb*) and *Saccharum spontaneum* (*Ss*).

Table S1 - 
*Cis*-regulatory elements in the regulatory region of *APX*, *APX-R* and *APX-L* genes from Poaceaes species.

Table S2 - Physicochemical parameters and subcellular predictions from *APX*, *APX-R* and *APX-L* genes in Poaceae species.
