# Supplementary material for: Genome-wide, evolutionary, and functional analyses of ascorbate peroxidase (APX) family in Poaceae species
Source: Genet Mol Biol. 2022 Dec 9;46(1 Suppl 1):e20220153. doi: 10.1590/1678-4685-GMB-2022-0153 (PMC9747090; doi:10.1590/1678-4685-GMB-2022-0153)
Supplement: Figure S1 - [file 1415-4757-GMB-46-1-s1-e20220153-s1.pdf]

Genetics and Molecular Biology

https://doi.org/10.1590/1678-4685-GMB-2022-0153

Supplementary Material to “Genome-wide, evolutionary, and functional analyses of ascorbate peroxidase (APX) family in Poaceae species”

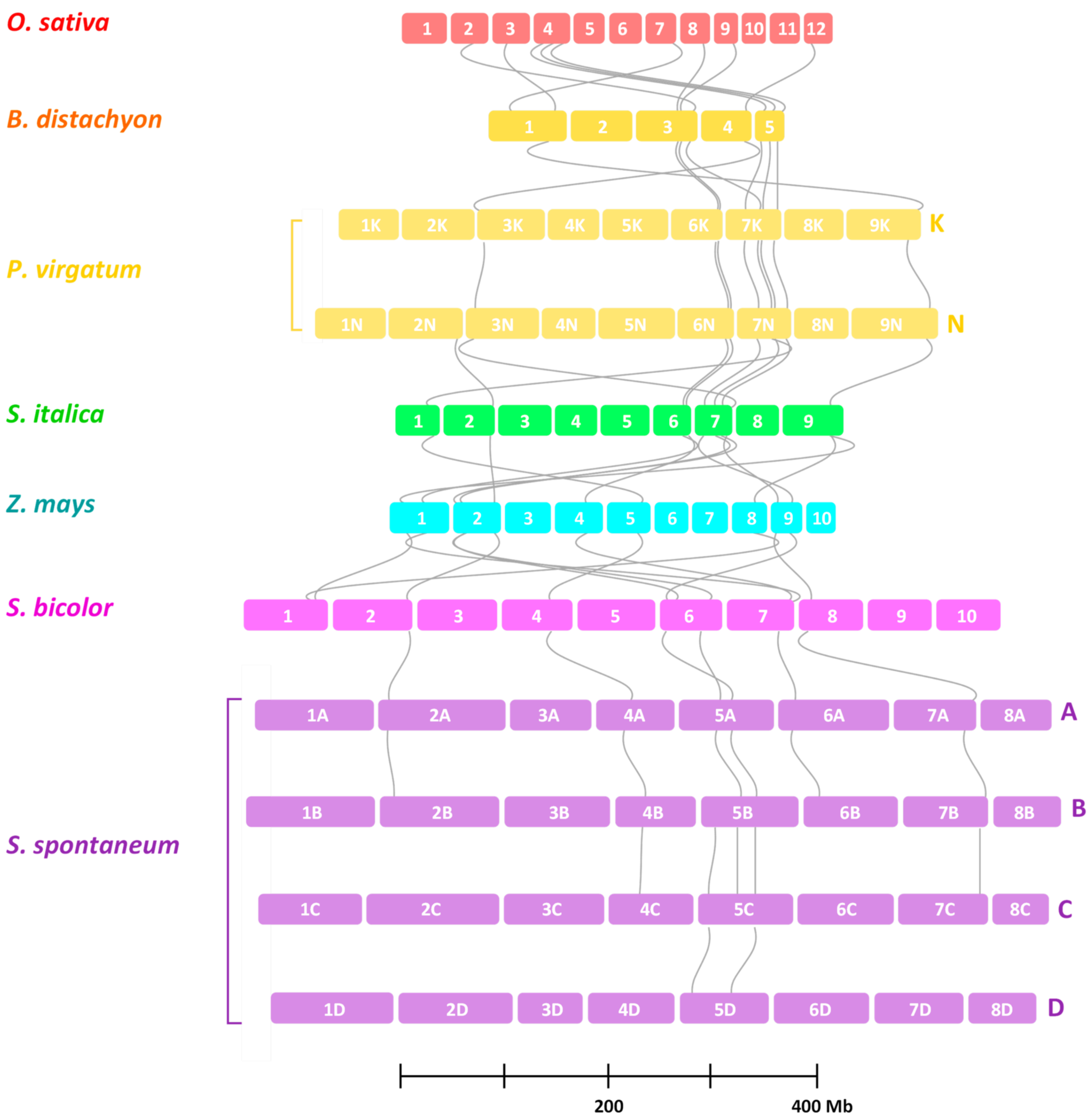

Figure S1 - Collinearity among APX, APX-R and APX-L genes in Oryza sativa, Brachypodium distachyion, Panicum virgatum (subgenomes K and N), Setaria italica, Zea mays, Sorghum bicolor and Saccharum spontaneum (subgenomes A, B, C and D). Chromosome numbers are displayed next to each bar. Gray lines indicate homologous gene pairs in the whole genome.
