# Supplementary material for: Genome-wide, evolutionary, and functional analyses of ascorbate peroxidase (APX) family in Poaceae species
Source: Genet Mol Biol. 2022 Dec 9;46(1 Suppl 1):e20220153. doi: 10.1590/1678-4685-GMB-2022-0153 (PMC9747090; doi:10.1590/1678-4685-GMB-2022-0153)
Supplement: Figure S3 - [file 1415-4757-GMB-46-1-s1-e20220153-s3.pdf]

Genetics and Molecular Biology

https://doi.org/10.1590/1678-4685-GMB-2022-0153

Supplementary Material to "Genome-wide, evolutionary, and functional analyses of ascorbate peroxidase (APX) family in Poaceae species"

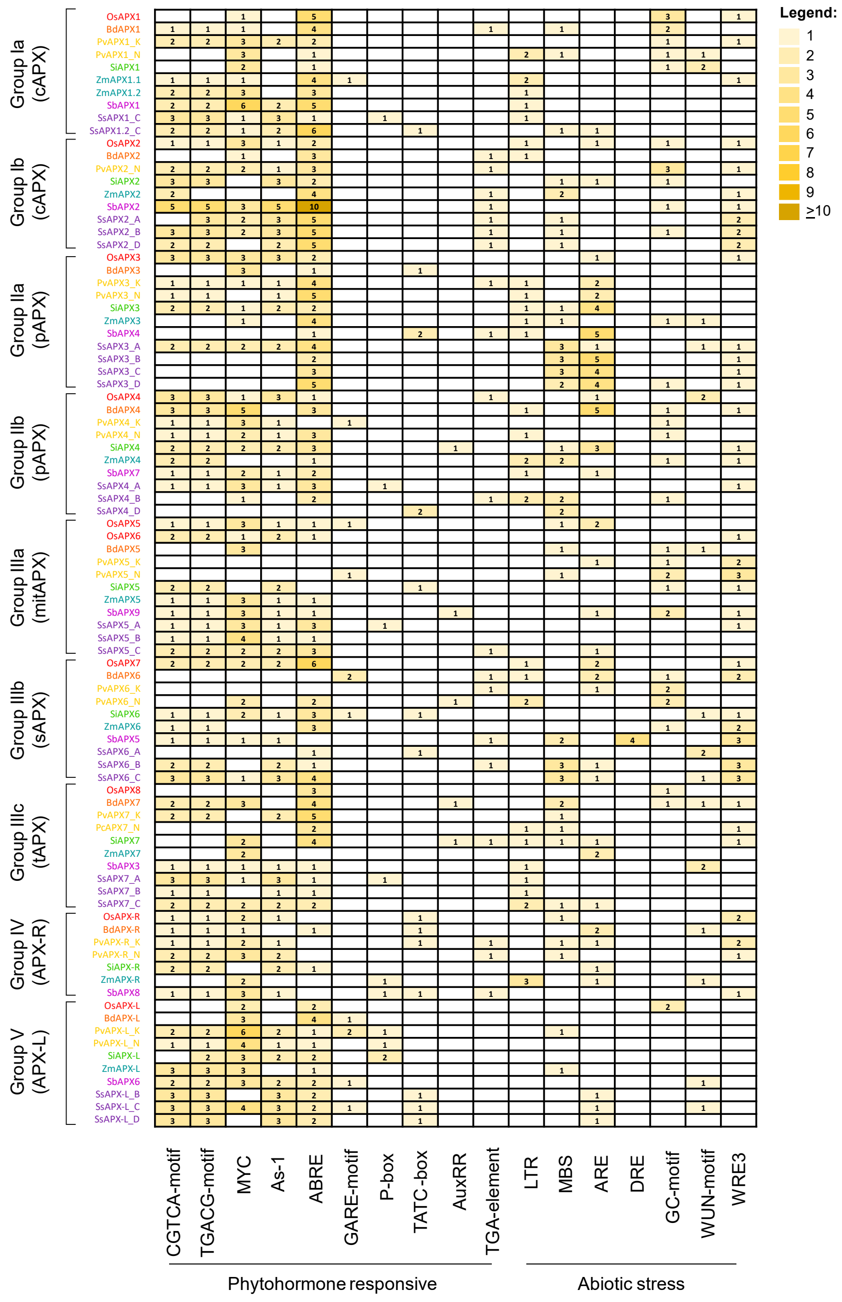

Figure S3 - Organization of cis-regulatory elements related to hormone responsiveness and environmental stress in *APX*, *APX-R* and *APX-L* genes from *Oryza sativa*, *Brachypodium distachyon*, *Panicum virgatum*, *Setaria italica*, *Zea mays*, *Sorghum bicolor* and *Saccharum spontaneum*. A 1-Kb promoter region of each gene was analyzed.
