# Supplementary material for: Genome-wide, evolutionary, and functional analyses of ascorbate peroxidase (APX) family in Poaceae species
Source: Genet Mol Biol. 2022 Dec 9;46(1 Suppl 1):e20220153. doi: 10.1590/1678-4685-GMB-2022-0153 (PMC9747090; doi:10.1590/1678-4685-GMB-2022-0153)
Supplement: Figure S4 - [file 1415-4757-GMB-46-1-s1-e20220153-s4.pdf]

Genetics and Molecular Biology

https://doi.org/10.1590/1678-4685-GMB-2022-0153

Supplementary Material to "Genome-wide, evolutionary, and functional analyses of ascorbate peroxidase (APX) family in Poaceae species"

Motif	Logo	E-value	Sites	Width
1	NCAPIMLRLAWHDAGTYDKNTKTGGP 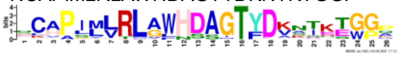	1.0e-1940	111	26
2	FYRMGLSDKDIVALSGAHTLGRAHPE 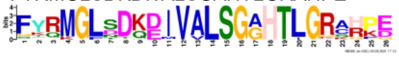	1.1e-1990	96	26
3	QLPTDKALLEDPSFRPYVEKYAEDZDAFFKDYAEAH 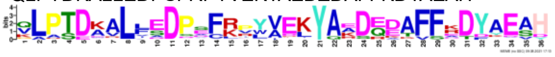	6.4e-2562	109	36
4	GAWTSEPLKFDNSYFKEJLEGEDEGL 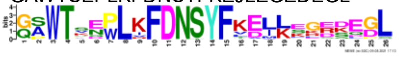	3.2e-1568	85	26
5	YADLYQLAGVVAVEVTGGPKIPFHPGRVD 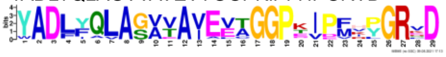	2.7e-1978	93	29
6	NGSIRFPAELKHGANAGLKIALKLJZPIKDKYPGIT 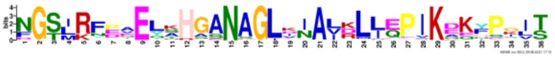	3.3e-2100	91	36
7	QCPPEGRLPDATKGSPHLR 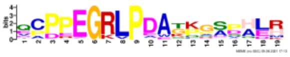	1.5e-1072	107	19
8	APVVDAEYLAQVEKARRDLRALIAEK 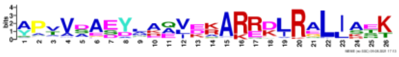	2.7e-767	75	26
9	RSGWGKPETKYTKBGPGAP 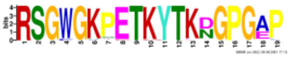	8.3e-529	36	19
10	AKLSELGFKF 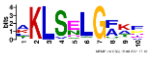	1.2e-443	108	10
11	ASAKSDLPTKALLAQAAFGVAVAAAVVAWAYLYEAN 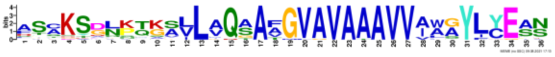	2.2e-406	24	36
12	YSYGKRELSDSMKQKIRAEYEGFGGSPDKPLQSNYFLNIMILIAGLAFLT 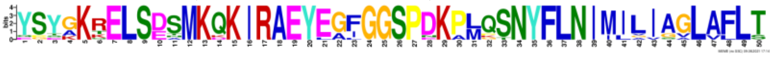	2.8e-381	12	50
13	QSALKRSFLDAAIAKCGGNEEKGRTLYSAYGSNGQWGLFDRTFGR 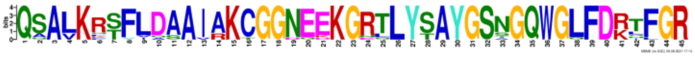	1.3e-380	13	45
14	NGSIIYEVDRPENSGLSRSJKILTKAKKEIDSIQKV 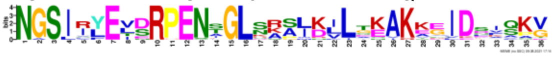	5.8e-273	18	36
15	GSTKFTGVATAADLIERRQRSEFQSSIKDTLYTAIK 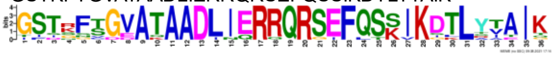	1.3e-188	11	36

Figure S4 - Sequence logos for the conserved motifs of APX, APX-R and APX-L proteins. The logos were identified by MEME software. The character and size of each logo represent the proportion of an amino acid at the specific site. The statistical analysis indicating the probability of obtaining the same alignment score in a random database of the same size and the same amino acid composition is indicated, as well as the frequency of the motifs in the set of proteins analyzed (sites) and the size of the motif (width).
