# Supplementary material for: Genome-wide, evolutionary, and functional analyses of ascorbate peroxidase (APX) family in Poaceae species
Source: Genet Mol Biol. 2022 Dec 9;46(1 Suppl 1):e20220153. doi: 10.1590/1678-4685-GMB-2022-0153 (PMC9747090; doi:10.1590/1678-4685-GMB-2022-0153)
Supplement: Figure S6 - [file 1415-4757-GMB-46-1-s1-e20220153-s6.pdf]

Genetics and Molecular Biology

https://doi.org/10.1590/1678-4685-GMB-2022-0153

Supplementary Material to "Genome-wide, evolutionary, and functional analyses of ascorbate peroxidase (APX) family in Poaceae species"

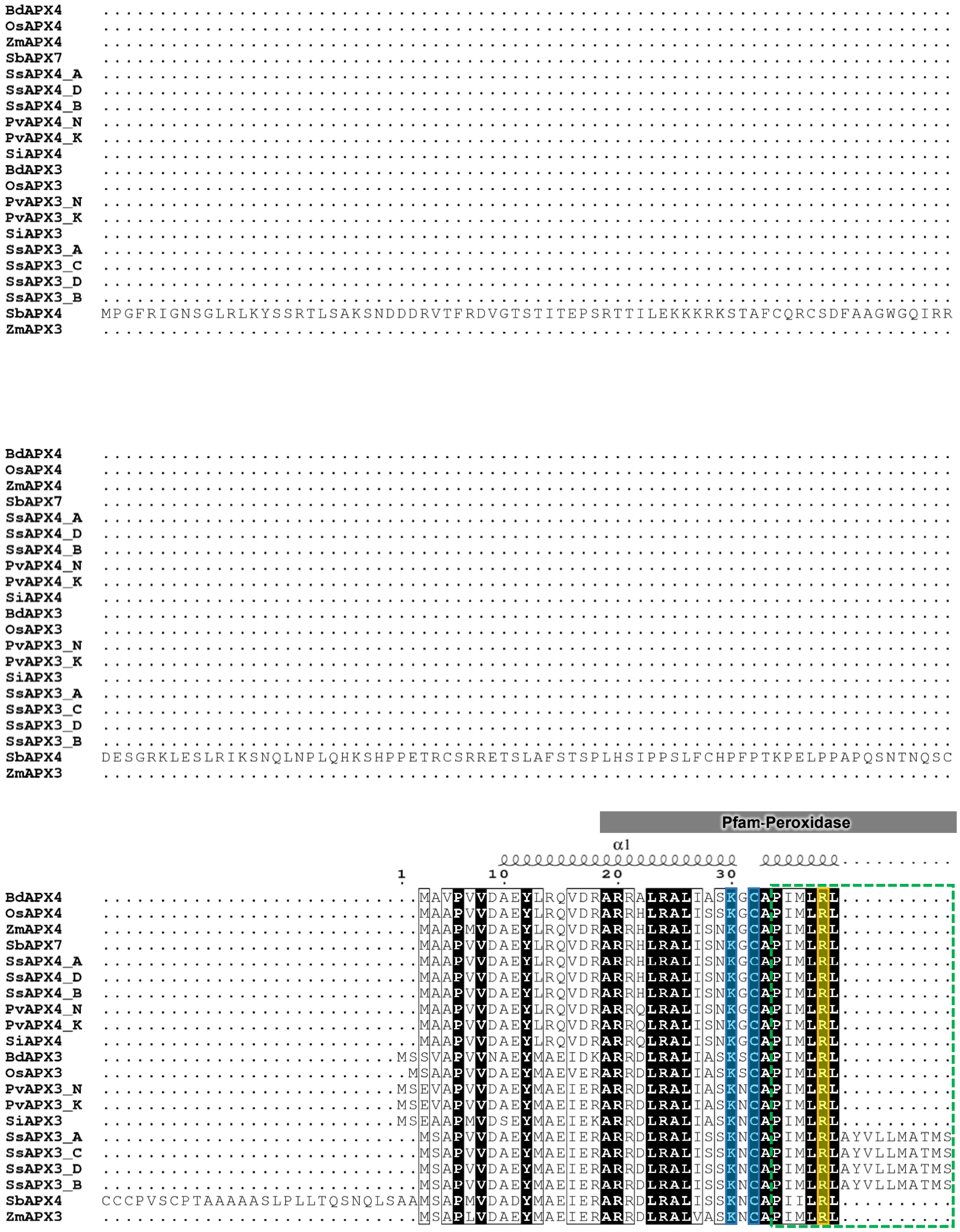

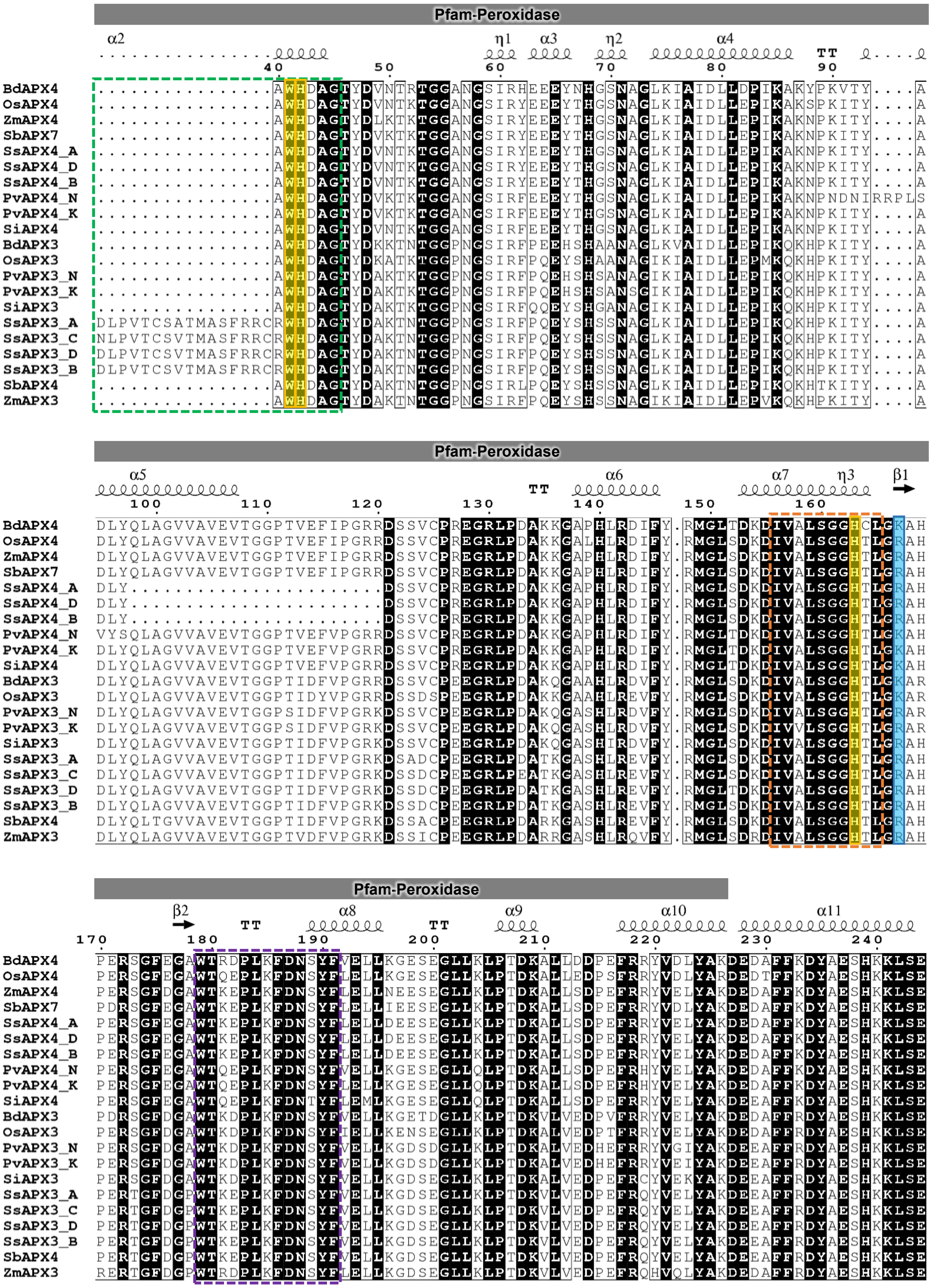
Pfam-Peroxidase
Pfam-Peroxidase
Pfam-Peroxidase
BdAPX4
OsAPX4
ZmAPX4
SbAPX7
SsAPX4_A
SsAPX4_D
SsAPX4_B
PvAPX4_N
PvAPX4_K
SiAPX4
BdAPX3
OsAPX3
PvAPX3_N
PvAPX3_K
SiAPX3
SsAPX3_A
SsAPX3_C
SsAPX3_D
SsAPX3_B
SbAPX4
ZmAPX3

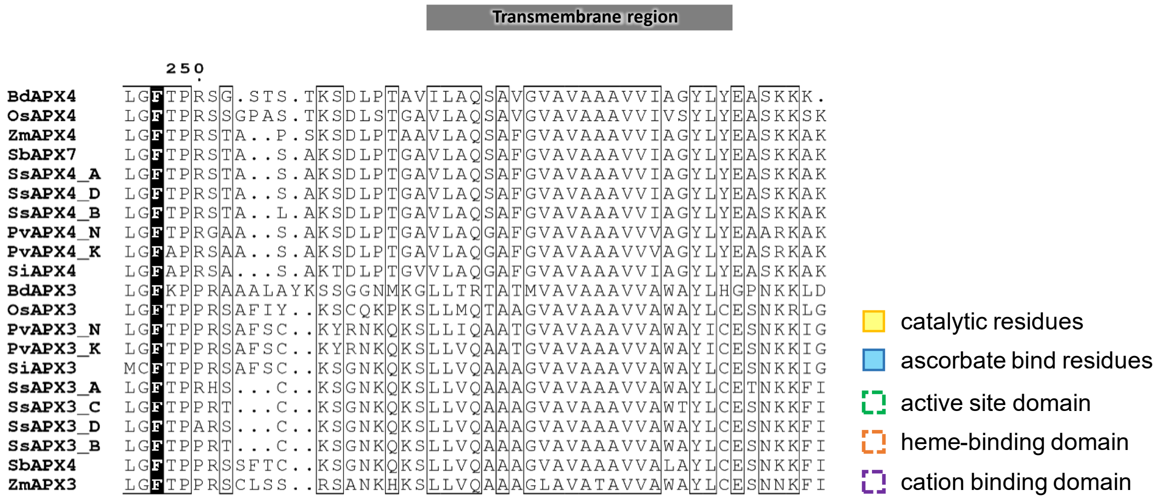

Figure S6 - Protein sequence alignment of peroxisomal APX (groups IIa and IIb) from *Oryza sativa* (*Os*), *Brachypodium distachyion* (*Bd*), *Panicum virgatum* (*Pv*), *Setaria italica* (*Si*), *Zea mays* (*Zm*), *Sorghum bicolor* (*Sb*) and *Saccharum spontaneum* (*Ss*). The deduced amino acid sequences of pAPX were aligned by Clustal Omega. Conserved amino acids are labeled in black.
