# Supplementary material for: Genome-wide, evolutionary, and functional analyses of ascorbate peroxidase (APX) family in Poaceae species
Source: Genet Mol Biol. 2022 Dec 9;46(1 Suppl 1):e20220153. doi: 10.1590/1678-4685-GMB-2022-0153 (PMC9747090; doi:10.1590/1678-4685-GMB-2022-0153)
Supplement: Figure S11 - [file 1415-4757-GMB-46-1-s1-e20220153-s11.pdf]

Genetics and Molecular Biology

https://doi.org/10.1590/1678-4685-GMB-2022-0153

Supplementary Material to "Genome-wide, evolutionary, and functional analyses of ascorbate peroxidase (APX) family in Poaceae species"

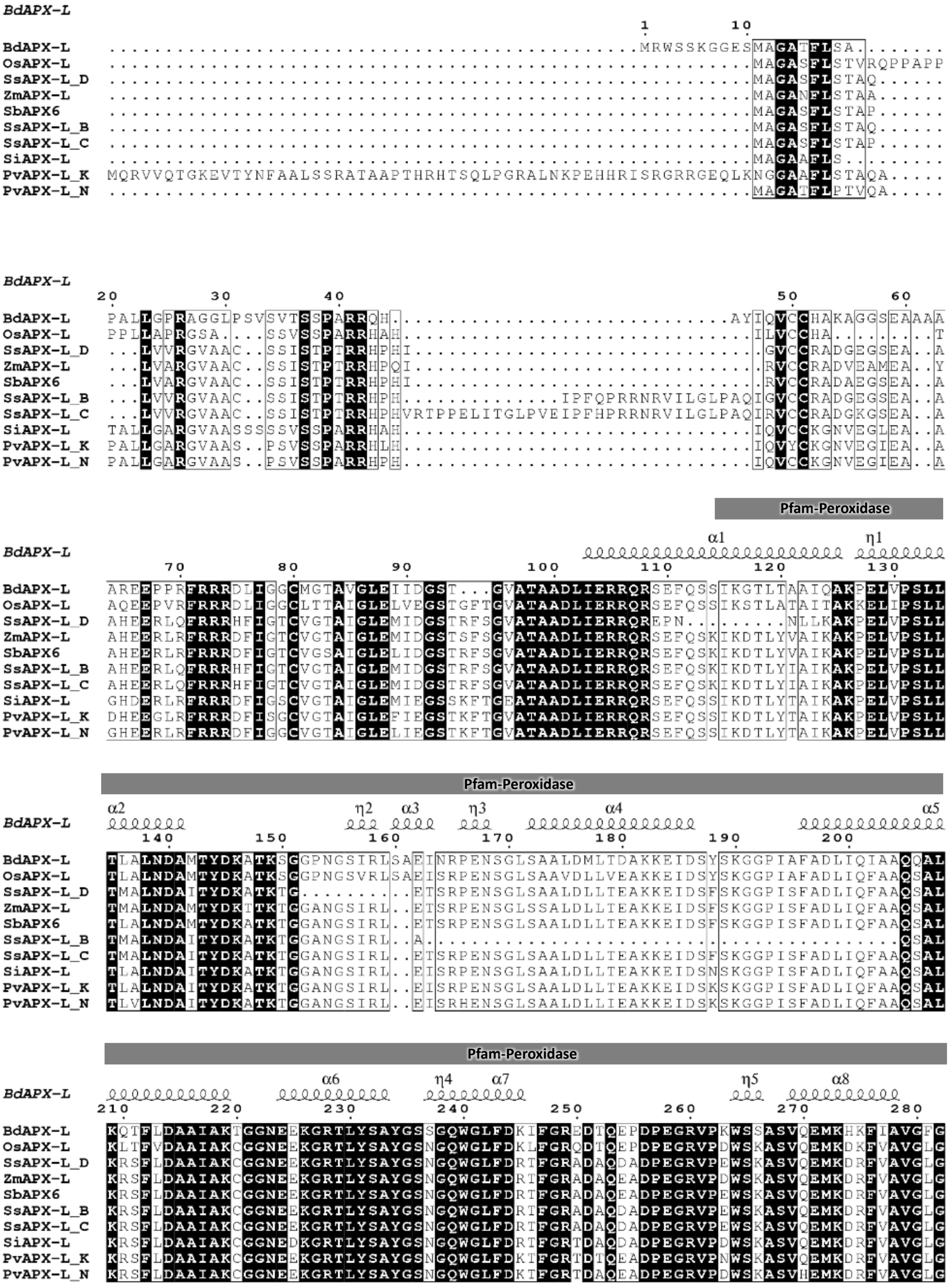

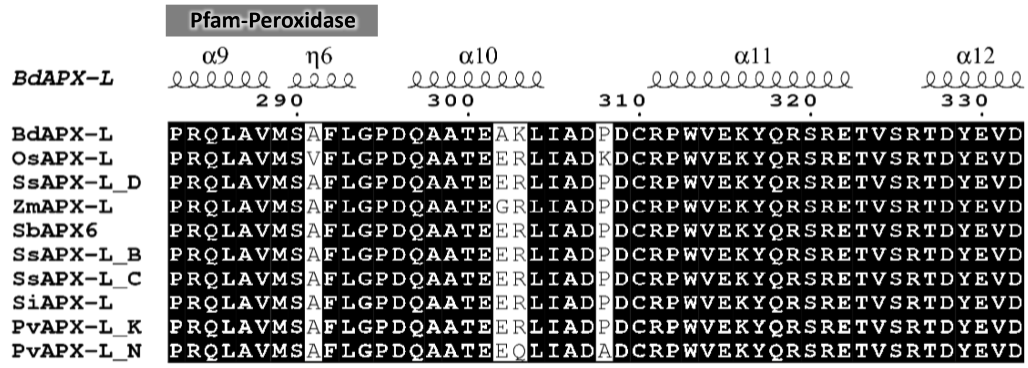

Figure S11 - Protein sequence alignment of APX-like (group V) from *Oryza sativa* (*Os*), *Brachypodium distachyion* (*Bd*), *Panicum virgatum* (*Pv*), *Setaria italica* (*Si*), *Zea mays* (*Zm*), *Sorghum bicolor* (*Sb*) and *Saccharum spontaneum* (*Ss*). The deduced amino acid sequences of APX-L were aligned by Clustal Omega. Conserved amino acids are labeled in black.
