# Supplementary material for: Genome-wide, evolutionary, and functional analyses of ascorbate peroxidase (APX) family in Poaceae species
Source: Genet Mol Biol. 2022 Dec 9;46(1 Suppl 1):e20220153. doi: 10.1590/1678-4685-GMB-2022-0153 (PMC9747090; doi:10.1590/1678-4685-GMB-2022-0153)
Supplement: Table S1 - [file 1415-4757-GMB-46-1-s1-e20220153-s12.pdf]

Genetics and Molecular Biology

https://doi.org/10.1590/1678-4685-GMB-2022-0153

Supplementary Material to "Genome-wide, evolutionary, and functional analyses of ascorbate peroxidase (APX) family in Poaceae species"

Table S1 - Cis-regulatory Elements in the Regulatory Region of APX, APX-R and APX-L genes from Poaceaes species.

https://1drv.ms/u/s!AiHfILIuSPrEg8Ysv9pQxJfRoHju8w?e=hrviil,
