# Supplementary material for: Genome-wide, evolutionary, and functional analyses of ascorbate peroxidase (APX) family in Poaceae species
Source: Genet Mol Biol. 2022 Dec 9;46(1 Suppl 1):e20220153. doi: 10.1590/1678-4685-GMB-2022-0153 (PMC9747090; doi:10.1590/1678-4685-GMB-2022-0153)
Supplement: Table S2 - [file 1415-4757-GMB-46-1-s1-e20220153-s13.pdf]

Genetics and Molecular Biology

https://doi.org/10.1590/1678-4685-GMB-2022-0153

Supplementary Material to "Genome-wide, evolutionary, and functional analyses of ascorbate peroxidase (APX) family in Poaceae species"

Table S2 - Physicochemical parameters and subcellular predictions from *APX*, *APX-R* and *APX-L* genes in Poaceae species. Gene name: proposed nomenclature; locus ID; phylogenetic group; size in aminoacid, Mass - molecular weight; pI - isoelectric point; GRAVY - grand average of hydropathy; Instability Index.

https://1drv.ms/u/s!AiHfILIuSPrEg8Ysv9pQxJfRoHju8w?e=hrviil
